# ANGPTL4 accelerates ovarian serous cystadenocarcinoma carcinogenesis and angiogenesis in the tumor microenvironment by activating the JAK2/STAT3 pathway and interacting with ESM1

**DOI:** 10.1186/s12967-023-04819-8

**Published:** 2024-01-11

**Authors:** Yu-kun Li, An-bo Gao, Tian Zeng, Dan Liu, Qun-feng Zhang, Xiao-min Ran, Zhen-zi Tang, Yan Li, Jue Liu, Ting Zhang, Gang-qing Shi, Wen-chao Zhou, Wen-da Zou, Juan Peng, Juan Zhang, Hui Li, Juan Zou

**Affiliations:** 1 Department of Assisted Reproductive Centre, Zhuzhou Central Hospital, Xiangya Hospital Zhuzhou Central South University, Central South University, Zhuzhou, Hunan China; 2 The Second Affiliated Hospital, Department of Gynecology, Hunan Province Key Laboratory of Tumor Cellular & Molecular Pathology, Cancer Research Institute, Hengyang Medical School, University of South China, Hengyang, Hunan China; 3 Clinical Research Institute, The Second Affiliated Hospital, Hengyang Medical School, University of South China, Hengyang, Hunan China; 4 Department of Gynecologic Oncology, School of Medicine, Hunan Cancer Hospital, The Affiliated Cancer Hospital of Xiangya, Central South University, Changsha, Hunan China

**Keywords:** Ovarian cancer, ANGPTL4, ESM1, Angiogenesis, JAK2/STAT3 pathway

## Abstract

**Background:**

Ovarian cancer (OC) is a malignant neoplasm that displays increased vascularization. Angiopoietin-like 4 (ANGPTL4) is a secreted glycoprotein that functions as a regulator of cell metabolism and angiogenesis and plays a critical role in tumorigenesis. However, the precise role of ANGPTL4 in the OC microenvironment, particularly its involvement in angiogenesis, has not been fully elucidated.

**Methods:**

The expression of ANGPTL4 was confirmed by bioinformatics and IHC in OC. The potential molecular mechanism of ANGPTL4 was measured by RNA-sequence. We used a series of molecular biological experiments to measure the ANGPTL4-JAK2-STAT3 and ANGPTL4-ESM1 axis in OC progression, including MTT, EdU, wound healing, transwell, xenograft model, oil red O staining, chick chorioallantoic membrane assay and zebrafish model. Moreover, the molecular mechanisms were confirmed by Western blot, Co-IP and molecular docking.

**Results:**

Our study demonstrates a significant upregulation of ANGPTL4 in OC specimens and its strong association with unfavorable prognosis. RNA-seq analysis affirms that ANGPTL4 facilitates OC development by driving JAK2-STAT3 signaling pathway activation. The interaction between ANGPTL4 and ESM1 promotes ANGPTL4 binding to lipoprotein lipase (LPL), thereby resulting in reprogrammed lipid metabolism and the promotion of OC cell proliferation, migration, and invasion. In the OC microenvironment, ESM1 may interfere with the binding of ANGPTL4 to integrin and vascular-endothelial cadherin (VE-Cad), which leads to stabilization of vascular integrity and ultimately promotes angiogenesis.

**Conclusion:**

Our findings underscore that ANGPTL4 promotes OC development via JAK signaling and induces angiogenesis in the tumor microenvironment through its interaction with ESM1.

**Supplementary Information:**

The online version contains supplementary material available at 10.1186/s12967-023-04819-8.

## Background

In cases of ovarian cancer (OC), there is multifocal intraperitoneal dissemination and ascitic fluid accumulation with intense neovascularization [1]. Angiogenesis is the physiologic process in which new blood vessels develop from preexisting vessels and is required for tumors beyond 1–2 mm in diameter. This progression is regulated by acidosis, hypoxia, and metabolic deficiency, resulting in angiogenesis that supplies oxygen and nutrients to tumors while removing metabolic waste products and promoting metastasis [2]. In clinical treatment, bevacizumab, an important antiangiogenic drug, improved progression-free survival in women with OC [3]. However, there is an addition to an increase in redundant angiogenic factors other than VEGF that results in the resistance to bevacizumab [4]. Therefore, it is urgent to confirm novel angiogenesis-related genes (ARGs) for OC patients, which may help distinguish OC patients at high risk, predict treatment prognosis and outcome, and even provide new therapeutic options.

Angiopoietin-like 4 (ANGPTL4), a glycosylated and secreted protein, contains a C-terminal ANGPTL4 fibrinogen domain (cANGPTL4) and N-terminal ANGPTL4 coiled-coil domain (nANGPTL4), which can regulate glucose and lipid metabolism [5]. cANGPTL4 is responsible for vascular disruption by interacting with vascular-endothelial cadherin (VE-Cad) and integrin α5β1 in endothelial cells [6]. nANGPTL4 can directly bind to lipoprotein lipases (LPLs) to repress their activities in hypertriglyceridemia [7, 8]. Recent studies have indicated that ANGPTL4 plays a contradictory role in tumor angiogenesis. ANGPTL4 can promote the development and progression of multiple cancer types, including breast cancer [9], pancreatic cancer [10], glioblastoma [11] and colon cancer [12]. Although these studies indicate a proangiogenic role for ANGPTL4 in cancers, an antiangiogenic role has also been suggested for ANGPTL4. In gastric cancer, Okochi-Takada and colleagues revealed that ANGPTL4 is a genetically and epigenetically inactivated secreted tumor suppressor in cancer angiogenesis [13]. Hui et al. found that ANGPTL4 knockdown could promote the migration of pancreatic cancer [14]. Ito and colleagues indicated that ANGPTL4 could impede the proliferation, chemotaxis and tube formation of endothelial cells [15]. These disagreements indicated that the effect of ANGPTL4 may be highly dependent on the molecular interactions in the tumor microenvironment of different tumor types and is closely related to the subcellular localization of its executive function.

Endothelial cell-specific molecule 1 (ESM1), a significant secreted protein, plays a key role in endothelium-dependent pathological disorders. The dysregulation of this secreted protein was observed in multiple cancer types, including metastatic prostate cancer [16], liver cancer [17], breast cancer [18], endometrial cancer and OC [19]. In our previous study, we found that ESM1 could significantly promote OC cell proliferation, apoptosis escape, migration, invasion, and angiogenesis by indirectly activating Akt signaling [20]. However, little is known regarding the direct molecular mechanism of ESM1 in OC progression.

Herein, this study aimed to confirm the role of ANGPTL4 in OC progression, especially in proliferation, invasion and angiogenesis, and to verify the effect of the interaction between ANGPTL4 and ESM1 on OC progression.

## Materials and methods

### Bioinformatic analysis

The list of ARGs was download from GSEA database (https://www.gsea-msigdb.org/gsea/index.jsp). The Human Gene Set: ANGIOGENESIS Genes annotated by the GO term GO:0001525 (https://www.gsea-msigdb.org/gsea/msigdb/human/geneset/ANGIOGENESIS.html) [21]. The expression, prognosis, and diagnosis of ARGs were confirmed by the TCGA database (https://portal.gdc.cancer.gov/) [22]. GO and KEGG enrichment were analyzed by the DAVID database (https://david.ncifcrf.gov/) [23]. ANGPTL4 protein was confirmed in the HPA database (https://www.proteinatlas.org/) [24]. LASSO regression, immune infiltration analysis, GSEA, GSVA and drug sensitivity analysis were performed according to our previous study [20, 25].

For LASSO regression, the RiskScore formula utilized in the model incorporates numerous genes, each assigned a weight. Negative values indicate the gene’s role as a protective factor, whereas positive values indicate its potential as a hazardous factor. Notably, the distinction between the Signature and nomogram prognostic models lies in their inclusion of genetic and clinical factors, respectively. Following the conversion of count data to transcripts per million (TPM) and subsequent normalization using the log2 (TPM + 1) method, the normalized transcription data were employed to calculate gene expression based on log2 (TPM + 1). Furthermore, the elimination of missing and incomplete samples occurred during the merging of clinical information. Consequently, a total of 376 ovarian serous cystadenocarcinoma (OSC) samples remained for subsequent analysis. The log-rank test was employed to compare the survival difference between groups. Additionally, the predictive accuracy of the risk score was assessed across all datasets through timedependent ROC curve analysis (v 0.4). The feature selection process employed the LASSO regression algorithm, while 10-fold cross-validation was utilized. The analysis was conducted using the R package glmnet. The R software package ggstatsplot was employed to depict the correlations between gene expression and immune score. Additionally, the visualization of differentially expressed genes was achieved using the “pheatmap” package in R software. The RiskScore formula was calculated as the sum of the expression amount of each gene multiplied by the corresponding coefficient (S). Based on the median risk score, the patients were categorized into two groups. All statistical analyses were conducted using the R package. A p value of less than 0.05 was deemed to be statistically significant.

For immune infiltration analysis, RNA sequencing expression profiles (level 3) and corresponding clinical information have been downloaded from the TCGA portal. Correlations between gene expression and immune score were drawn with the ggstatsplot package of R software, multi-gene correlations were drawn with the pheatmap package of R software. Spearman’s correlation analysis was used to describe correlation between quantitative variables without a normal distribution.

For GSEA analysis, GSEA employs a predetermined gene set derived from the KEGG database to arrange genes based on their varying expression levels in the two distinct sample types. Subsequently, it examines whether the pre-established gene set exhibits enrichment at either the uppermost or lowermost positions within the sequencing table. This study employed Gene Set Enrichment Analysis (GSEA) to assess the disparities in signaling pathways between the high and low expression cohorts of core genes. The objective was to investigate the potential molecular mechanisms underlying the contrasting prognoses observed in these two groups, while also offering insights into the involvement of core genes in tumor progression.

In order to assess the enrichment of transcriptional gene sets, gene set variation analysis (GSVA) is employed as a nonparametric, unsupervised approach. In order to comprehensively score the genes under investigation, it is possible to determine the biological function of a sample by transforming gene-level alterations into pathway-level changes using GSVA. Based on the Molecular Signatures Database gene sets were obtained for this study, and each gene set was scored using the GSVA algorithm, which enables it to be evaluated for potential alterations in the biological function over time.

For drug sensitivity analysis, we used GSCA database (http://bioinfo.life.hust.edu.cn/GSCA/#/) to confirm the correlation between ANGPTL4 mRNA level and multiple drugs [26].

### Clinical sample collection

Thirty-five ovarian cancer and corresponding para-carcinoma tissue samples, 20 ovarian granular cell carcinoma and corresponding para-carcinoma tissue samples, 15 teratoma samples, 20 ovarian mucinous cystadenoma samples, and 20 normal ovary samples were collected from Zhuzhou Central Hospital, Xiangya Hospital Zhuzhou Central South University, Central South University from 2018 to 2022 with ethics standards of the Helsinki Declaration. None of the patients underwent radiotherapy or chemotherapy. This study was approved by the ethics committee of Central South University.

### Cell culture and treatment

OC cells (Hey-A8, and SKOV3, CVCL_0532) and human umbilical vein endothelial cells (HUVECs) were purchased from ATCC (Manassas, USA). These cells were cultured in McCoy’s 5 A (Shanghai Zhong Qiao Xin Zhou Biotechnology Co., Ltd, ZQ-1000) (SKOV3) or DMEM (Shanghai Zhong Qiao Xin Zhou Biotechnology Co., Ltd, ZQ-100) (HUVECs and Hey-A8) with 10% FBS (Shanghai Zhong Qiao Xin Zhou Biotechnology Co., Ltd, ZQ500-A) and 1% PS (Shanghai Zhong Qiao Xin Zhou Biotechnology Co., Ltd, CSP006). ANGPTL4 shRNA, ANGPTL4 OE plasmid, and ESM1 OE plasmid were purchased from HonorGene (Changsha, China). For cell transfection, these shRNAs or plasmids were transfected into the Hey-A8 and SKOV3 cell lines with Lipofectamine® 3000 (Thermo Fisher Scientific, Inc.) according to the manufacturer’s instructions.

### Cell function experiments

3-(4,5-dimethylthiazol-2-yl)-2,5-diphenyltetrazolium bromide (MTT) analysis, 5-ethynyl-2′-deoxyuridine (Edu) analysis, wound healing analysis, transwell assay, and tube formation analysis were performed following the protocols in our previous study [20]. MTT involved the introduction of 5 × 10^3^ cells per well into 96-well plates, followed by an incubation period of 1, 2, and 3 days at a temperature of 37 degrees Celsius. An incubation period of four hours at 37 degrees Celsius was followed by the addition of 20 µl of MTT solution (5 mg/ml, Sigma-Aldrich; Merck KGaA). Following that, 150 ml of DMSO was introduced in order to dissolve the precipitates. Using a microplate reader (Molecular Devices, LLC), we evaluated the impact of cell quantity in terms of absorbance at 490 nm. As part of the EdU assay, RiboBio’s EdU kit (Guangzhou, China) was used to evaluate cell proliferation. In the wound healing assay, a total of 5 × 10^5^ cells were initially seeded in 6-well plates. Subsequently, the plates were scratched using a pipette tip and rinsed with 1640/DMEM solution. The cells were then incubated at a temperature of 37 degrees Celsius for a duration of 24 h. Photographic documentation was captured at both the 0 and 24-hour time points. For the Transwell assay, a quantity of 2.5 × 10^5^ cells were seeded in a 24-well Transwell chamber (Costar, Cambridge, MA). This chamber was utilized for both the invasion and migration assays, with or without the presence of Matrigel. The cells were cultured in 1640 medium supplemented with 10% FBS for a period of 16 h, maintaining a temperature of 37 degrees Celsius and a CO_2_ concentration of 5%. Finally, the cells that had migrated or invaded the lower surface of the filter were stained using crystal violet. For tube formation analysis, Matrigel (200 µl) was introduced into every well of a 48-well plate, followed by the seeding of 1 × 10^4^ primary HUVECs in 50 µl of conditioned medium obtained from OC cells. Subsequently, the plate was incubated at 37 degrees Celsius for 6 h, and images were captured utilizing a fluorescence microscope.

### Western blot

An appropriate amount of trypsin was added to the container for cultivating cells, digested for an appropriate time, and collected and centrifuged to obtain the cells. Next, the centrifuged cells were transferred to ice, washed with precooled phosphate buffered saline (PBS), and recentrifuged, repeating the process twice to obtain clean cells. The cells were then fully mixed with RIPA lysis buffer (Yoche, YSD0100) containing 1% 1 mM phenylmethylsulfonyl fluoride (PMSF) (Bioss, C05-02001) and placed on ice for 30 min. After the cells were fully lysed, the mixture was centrifuged to obtain cell fragments and supernatants containing cellular proteins, which were carefully absorbed and transferred to a clean container and placed on ice. Then, the protein concentration was determined by the Bradford method. According to the specifications of the BCA protein quantitative kit (Yoche, YSD-500T), different concentrations of protein standard which was prepared using bovine serum albumin (BSA) were used as the standard control to measure the corresponding absorbance at 562 nm and to draw the standard absorbance-concentration curve. Then, an appropriate amount of protein supernatant sample was absorbed, the absorbance was measured under the same conditions, and the protein concentration of the sample was converted according to the previous standard curve. After the concentration was determined, the protein sample and SDS‒PAGE loading buffer (Bioss, C05-03001) were mixed at 4:1 and heated at 100 °C for 5 min, which fully denatured the protein. The protein mixture was cooled to room temperature and centrifuged at 12,000 rpm for 5 min. An appropriate amount of the sample was added to a 10% SDS gel, electrophoresed to 1 cm near the bottom of the gel, transferred to a polyvinylidene difluoride (PVDF) membrane, and incubated with 5% skim milk at room temperature for 3 h. Next, the PVDF membrane was transferred to the primary antibody and shaken back and forth at 4 °C for 12 h. The membrane was then transferred to the secondary antibody and reacted at room temperature for 2 h. After completing the previous steps, the PVDF membrane was washed with tris buffered saline with tween-20 (TBST) for 5 min three times. Finally, the membrane was analyzed by chemiluminescence with Superkine West Femto Maximum Sensitivity Substrate (Abbkine, BMU102-CN). The primary antibodies used are shown below: ESM1 (Abcam, ab103590), STAT3 (Abcam, ab68153), p-STAT3 (Abcam, ab267373), JAK2 (Abcam, ab108596), p-JAK2 (Abcam, ab32101), Integrin α5β1 (Abcam, ab239400), VE-Cad (Abcam, ab205336), hypoxia-inducible factor 1alpha (HIF-1α) (Abcam, ab51608), proliferating cell nuclear antigen (PCNA) (Abcam, ab265609), and vimentin (Abcam, ab92547). and β-actin (Abcam, ab8226).

### Immunofluorescence staining (IF)

Cells grown on glass coverslips were fixed with 4% paraformaldehyde and permeabilized with 0.3% Triton X-100. After blocking with BSA, cells were incubated with primary antibody at 4 °C overnight followed by incubation with fluorescent secondary antibody for 2 h at room temperature. 4’,6-diamidino-2-phenylindole (DAPI) (0.1%) was used to label the nucleus for 15 min. Cells were examined under a confocal laser microscope (Leica TCS SP5II STED, Mannheim, Germany). At least 200 cells on each slide were counted, and the percentage of cells with targeting protein was calculated. The primary antibodies used are shown below: ANGPTL4 (Abcam, ab196746), ESM1 (Abcam, ab103590) and CD34 (Abcam, ab81289).

### Immunohistochemistry staining (IHC)

Immunohistochemical examination was performed with a two-step detection kit (ZSBG-BIO, PV9000). First, the pathological tissue was soaked in three fresh xylenes in sequence for 20 min each. After the excess liquid was removed, the slices were soaked in anhydrous ethanol, 95% ethanol, and 75% ethanol for six minutes each. Tissue sections were then rinsed with distilled water for 1 min and placed in PBS. The sections were placed in citric acid repair solution (pH 6.0) under high temperature and pressure for the repair of antigen sites in the tissue sections for 15 min. After cooling to room temperature, the sections were incubated with the appropriate endogenous peroxidase blocker at 37 °C for 20 min and then washed with PBS buffer solution 3 times. Sections were then incubated with primary antibody at 37 °C for 2 h and washed with PBS 3 times. The tissues were then dripped with appropriate reaction enhancers, incubated at 37 °C for 20 min, and cleaned with PBS 3 times. The tissues were then allowed to bind to the secondary antibodies and washed three times with PBS when they were incubated at room temperature for 20 min. A 3,3′-diaminobenzidine (DAB) color development kit (ZSBg-BIO, ZLI-9018) was then used for color development of tissue sections. Finally, the sections were stained with hematoxylin and dehydrated. The primary antibodies used are shown below: ANGPTL4 (Abcam, ab196746), PCNA (Abcam, ab265609), MMP9 (Abcam, ab76003), ESM1 (Abcam, ab103590) and vimentin (Abcam, ab92547). The staining of intensity was scored as follow: Negative, 0; weak to moderate, 1; strong, 2. The staining of quantity was scored as follow: None, 0; <25% and 75%–25%, 1; >75%, 2. The final score was obtained by adding the quantity score and intensity score. The score ≥ 2 and < 2 were defined as high and low expression.

### Oil red O staining

The improved oil red O staining solution was obtained by thoroughly shaking oil red O staining A and oil red O staining B (Abiowell, AW10570), mixing at 3:2 and allowing to stand for 10 min. Cells cultured in a six well plate were washed three times with PBS and fixed in 3.7% formaldehyde for 30 min before staining. After washing the fixed cells with distilled water, they were treated with 60% isopropanol for 30 s (sec), and then stained with oil red O staining solution at room temperature and in darkness for 12 min. Next, the cells were washed slightly in 60% isopropanol for approximately 7 s to remove the dye solution. Afterwards, the cells were washed twice with distilled water and then used for photography and counting. Finally, ImageJ was used to measure the length of lipid droplets in 100 cells, and the length of lipid droplets was measured in pixels.

### Coimmunoprecipitation (Co-IP)

Protein A/G agarose (Santa Cruz Biotechnology, USA) was utilized for coimmunoprecipitation. The pellets were washed three times with RIPA buffer and subjected to Western blot analysis to detect immunoprecipitated proteins.

### Glutathione-S-transferase (GST) pull-down

GST-fused ANGPTL4 was expressed and purified using standard protocols in BL21 E. coli with glutathione-Sepharose 4B beads. Coomassie blue staining was used for protein expression quantification. Cell lysates were mixed with purified GST-ANGPTL4 or GST-only beads and washed before immunoblotting. Ni Sepharose High-Performance histidine-tagged protein purification resin was used with 1 lg of His-rhESM1 and GST-ANGPTL4 at 4 °C overnight, followed by washing and SDS‒PAGE detection with the indicated antibody.

### Molecular docking

Molecular docking is used to verify the binding activity of protein‒protein or protein-small molecules. The HDOCK online website (http://hdock.phys.hust.edu.cn/) was used in this study for the molecular docking process. HDOCK can analyze different conformations of protein‒protein docking, binding activity under different conformations, and amino acid residues within 5 A of the interaction distance. The predicted three-dimensional structures of the basal proteins ESM1, ANGPTL4 and LPL were obtained from the Alphafold protein database. PyMOL (version 4.3.0) software was also used to map amino acid residues that interact between two proteins. Discovery Studio software for hydrogen bonding and hydrophobic forces between two proteins.

### RT-PCR analysis

The treated cells are grown to a suitable density. After washing the cells once with PBS buffer, 1 ml of Trizol (Vazyme, R401-01) was added to the cells of each treatment group and qualified RNA was extracted according to the instructions. Then reverse transcription kit (Vazyme, R223) was used to reverse transcribed the RNA obtained in the previous step according to the experimental steps to obtain corresponding cDNA. After obtaining the cDNA, the cDNA, ddH2O, Primer of the corresponding gene and 2×Rapid Taq Master Mix were mixed and reacted in Eppendorf Mastercycler X40 according to the instructions of the rapid PCR reaction reagent (Vazyme, P222). Finally, the obtained products were subjected to 2% agarose gel electrophoresis and the experimental results were analyzed.

The primer sequences were as follows: ANGPTL4 Forward: 5′-TCACAGCCTGCAGACACAACT-3′ Reverse: 5′-CCATCTCGGGCAGCCTCTTT-3′; ESM1 Forward: 5′-CCTTCGGGATGGATTGCAGA-3′ Reverse: 5′-CCGGCAGCATTCTCTTTCAC-3′; β-actin Forward: 5′-CCTGGCACCCAGCACAAT-3′ Reverse: 5′-GGGCCGGACTCGTCATAC-3′.

### RNA-sequence

According to the requirements of Beijing Genomics institution (BGI), the samples of Hey-A8 cells overexpressing ANGPTL4 and transfected with vector were broken and lysed, and RNA was purified and collected. Then, the samples were treated with sample quality control, mRNA isolation, mRNA fragmentation, cDNA synthesis, end repair, adaptor ligation, PCR, library quality control, circularization and sequencing. For details, please refer to the guide of BGI Corporation (https://www.yuque.com/yangyulan-ayaeq/oupzan/lmx609).

### Xenograft model

The 4 weeks old female athymic BALB/c nude mice (n = 5 for each group) were injected with OC cells (1 × 10^6^). Tumor volume was measured every seven days using the formula width^2^ × length × 0.5, and xenografts were removed, weighed, dehydrated, embedded in paraffin, and sectioned for staining after sacrificing the mice on Day 48.

### Chick embryo chorioallantoic membrane (CAM) assay

Fertilized chicken eggs were initially incubated in an 80% humidity environment at 37 °C for seven days. On the eighth day, a square opening was made on the eggshell to expose the CAM and covered with a gelatin sponge (0.3 cm × 0.3 cm × 0.3 cm) containing either PBS or conditioned medium (CM) Then, OC cells (2 × 10^5^/100 µl) were seeded onto Matrigel-coated chick embryo eggs and incubated for 4 days. The window was then taped and incubated for an additional 2 days before quantifying angiogenesis by counting blood vessel branches.

### Zebrafish model

Transgenic zebrafish embryos carrying Tg(FLK1:EGFP) were microinjected with approximately 600 °C cells per embryo (labeled with CellTracker™ CM-DiI, 1 µg/µl) at 48 h post fertilization to establish a zebrafish model for tumor cell transplantation (n = 3 for each group). The transplanted zebrafish were cultured in a 34 °C light incubator with a photoperiod of 14 h light and 10 h dark. Juvenile fish with the same number of tumor cells were cultured 24 h after transplantation. Confocal microscopy was used to capture photos two days later.

### Statistical analysis

All results were analyzed by t tests and one-way ANOVA with R language (Version 3.6). Each assay contained three biological replicates. A *P* < 0.05 was considered statistically significant.

## Results

### The expression and DNA alteration of ARGs in OC patients

**Fig. 1:**
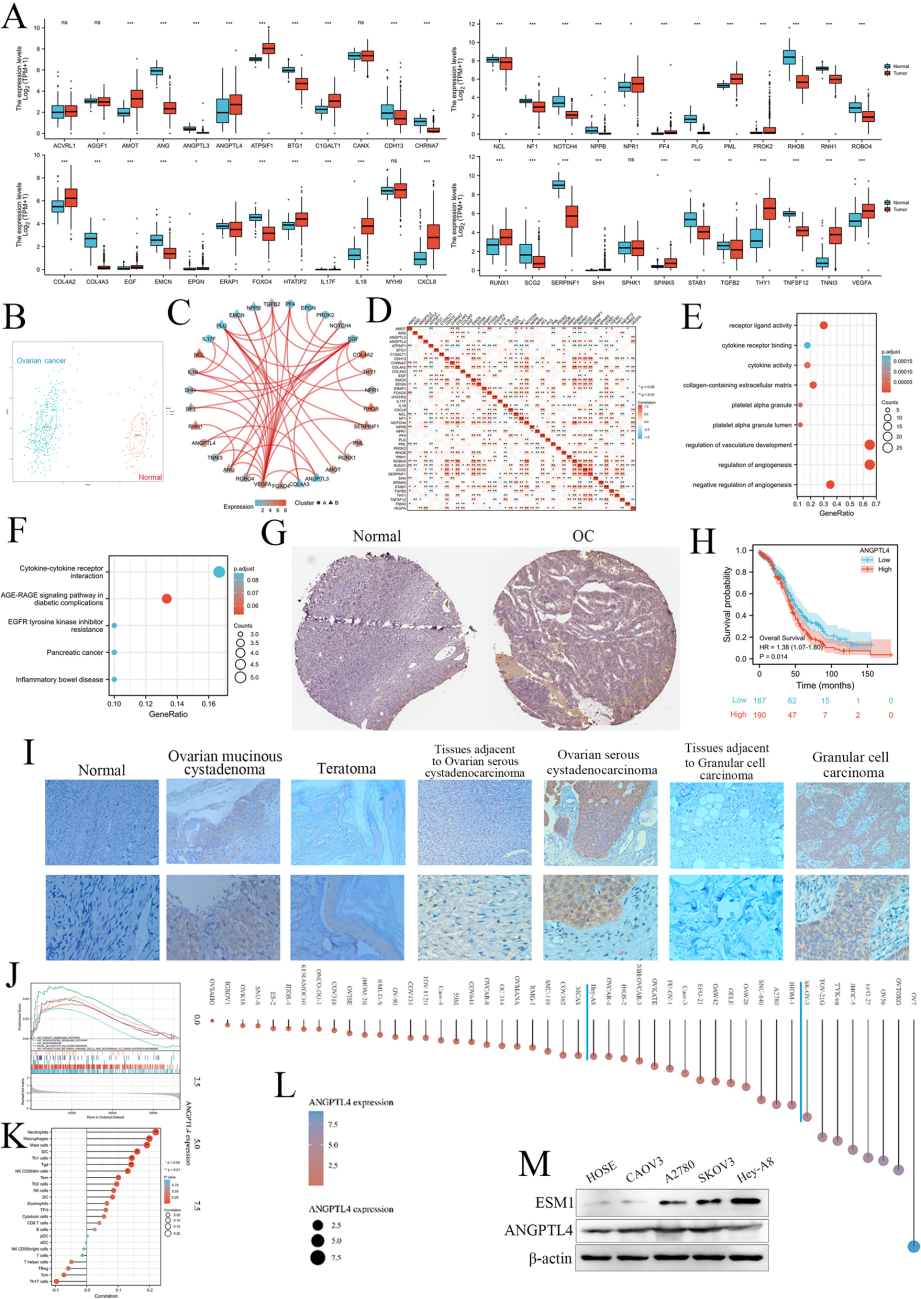
The expression, potential functions, diagnostic value and prognostic value of ARGs in OC patients. **A** The mRNA expression of ARGs in OC samples compared to normal ovary samples based on the TCGA database. **B** The diagnostic value of ARGs for OC patients. **C** The PPI network for ARGs. **D** The correlation among these ARGs in OC patients. **E** GO and **F** KEGG enrichment analysis for these ARGs. **G** ANGPTL4 protein expression in OC and normal ovaries based on the HPA database. **H** The prognostic value of ANGPTL4 for OC patients based on the TCGA database. **I** The expression of ANGPTL4 in normal ovary samples and benign and malignant ovarian disease samples. **J** GSEA of ANGPTL4 based on the TCGA database. **K** Immune infiltration analysis for OC patients according to ANGPTL4 expression. **L** The expression of ANGPTL4 in multiple OC cell lines. **M** The expression of ESM1 and ANGPTL4 in multiple OC cell lines by Western blot. *P < 0.05, **P < 0.01, ***P < 0.001

First, we confirmed the mRNA levels of these ARGs in OC tissue samples and normal ovary tissue samples based on the TCGA and GTEX databases, which showed that AMOT, ANGPTL4, ATP5IF1, C1GALT1, NPR1, PF4, PML, PROK2, COL4A2, EGF, HTATIP2, IL17F, IL18, CXCL8, RUNX1, SHH, SPINK5, THY1, TNNI3 and VEGFA were significantly increased in OC samples, but ANG, ANGPTL3, BTG1, CDH13, CHRNA7, NCL, NF1, NOTCH4, PLG, RHOB, RNH1, ROBO4, COL4A3, EMCN, ERAP1, FOXO4, SCG2, SERPINF1, STAB1, TGFB2, and TNFSF12 were significantly decreased in OC samples compared to normal ovary samples (Fig. 1A). Moreover, PCA indicated that these significantly differentially expressed ARGs had significant diagnostic value for distinguishing OC patients and normal women (Fig. 1B). The protein-protein interaction (PPI) network and correlation analysis both indicated that 31 hub genes might have close coexpression and interaction in OC patients (Fig. 1C, D). GO and KEGG analyses found that these ARGs were enriched in receptor ligand activity, cytokine receptor binding, regulation of angiogenesis, cytokine‒cytokine receptor interaction, advanced glycation end product (AGE)-receptor for AGE (RAGE) signaling pathway in diabetic complications, and EGFR tyrosine kinase inhibitor resistance (Fig. 1E, F).

### The prognostic significance of ARGs in OC patients

Moreover, we constructed a risk model by least absolute shrinkage and selection operator (LASSO) regression (lambda.min = 0.0416), and 13 signatures were confirmed (Additional file 1: Fig. S1A, B). Risk score = (0.1055)*ANGPTL4 +(− 0.0504)*ATP5IF1 +(− 0.0374)* (CHRNA7) +(− 0.0308)*COL4A3 +(− 0.1499)*EGF +(− 0.0897)*NOTCH4 +(− 0.0124)*PF4 +(0.6701)*PLG +(0.0409)*RUNX1 +(− 0.0359)*SHH +(0.0583)*STAB1 +(0.0332)*THY1 +(− 0.0622)*VEGFA. OC patients were divided into high-risk and low-risk groups by the risk score (The cutoff value was 0.0149, high-risk ≥ 0.0149 and low-risk < 0.0149) (Additional file 1: Fig. S1C). Survival analysis indicated that OC patients in the high-risk group had a significantly poorer prognosis than low-risk OC patients (Additional file 1: Fig. S1D). The area under the curve (AUC) values at 1, 3, and 5 years were 0.598, 0.626, and 0.671, respectively (Additional file 1: Fig. S1E). Furthermore, the risk score might correlate with immune cell infiltration, especially in CD4 T cells, neutrophils, macrophages, and myeloid dendritic cells (Additional file 1: Fig. S1F). The Multi-Cox and Uni-Cox analyses both indicated that ANGPTL4, VEGFA and ATP5IF1 are hub risk ARGs for OC patients, which indicated that these 3 genes have a more significant prognostic value compared to other signatures (Additional file 1: Fig. S2A, B). We further combined the mRNA levels of these ARGs to construct a nomogram to predict the survival probability of patients at 1, 3, and 5 years for Osteosarcoma (OS). The nomogram suggested that the prognostic prediction of the mRNA level of ANGPTL4 was better than that of RUNX1 in OS (Additional file 1: Fig. S2C, D). Drug sensitivity analysis showed that the mRNA expression of ANGPTL4 was obviously and negatively regulated by multiple anti-angiogenesis drugs (piperlongumine, BMS-708,163, and Methylstat), which indicated that ANGPTL4 was a key drug susceptibility ARG in OC patients (Additional file 1: Fig. S2E, F). Moreover, PCR analysis showed that anti-angiogenic drugs, bevacizumab, could enhance the mRNA level of ESM1 and ANGPTL4 in SKOV3 cell lines (Additional file 1: Fig. S2G). Taken together, these results indicated that ANGPTL4 might be a hub ARG in OC development.

#### ANGPTL4 as a hub ARG in the development of OC

ANGPTL4 was frequently and significantly dysregulated in multiple cancer types, including Adrenocortical carcinoma (ACC), Breast invasive carcinoma (BRCA), Cholangio carcinoma (CHOL), Colon adenocarcinoma (COAD), Lymphoid Neoplasm Diffuse Large B-cell Lymphoma (DLBC), Esophageal carcinoma (ESCA), Glioblastoma multiforme (GBM), Kidney Chromophobe (KICH), Kidney renal clear cell carcinoma (KIRC), Liver hepatocellular carcinoma (LIHC), Lung squamous cell carcinoma (LUSC), OC, Pancreatic adenocarcinoma (PAAD), Prostate adenocarcinoma (PRAD), Rectum adenocarcinoma (READ), Skin Cutaneous Melanoma (SKCM), Stomach adenocarcinoma (STAD), Testicular Germ Cell Tumors (TGCT), Thymoma (THYM), Uterine Corpus Endometrial Carcinoma (UCEC), and Uterine Carcinosarcoma (UCS) (Additional file 1: Fig. S3). To further elucidate the role of ANGPTL4 in OC progression, we confirmed its protein expression in the HPA database for OC patients, which showed that ANGPTL4 expression was obviously increased in OC patients (Fig. 1G; Table 1). ANGPTL4 was also significantly correlated with poor prognosis in OC patients based on the TCGA database (Fig. 1H). Therefore, we detected the expression in our OC dataset based on IHC staining. We found that the expression of ANGPTL4 was significantly increased in benign ovarian disease (ovarian mucinous cystadenoma and teratoma) and OC (ovarian serous cystadenocarcinoma and granular cell carcinoma) compared to normal ovary samples and corresponding para-carcinoma tissue samples (Fig. 1I; Table 2). The ANGPTL4 mRNA level was significantly and positively correlated pro-angiogenic factors based on TCGA database OC dataset, such as ESM1 and VEGFA (Table 3). GSEA indicated that ANGPTL4 was correlated with the phosphatidylinositol 3-kinase (PI3K)—serine/threonine-protein kinases (Akt) pathway, VEGFA/vascular endothelial growth factor receptor 2 (VEGFR2) pathway, angiogenesis, glycolysis, and the interaction between immune cells and microRNAs in the tumor microenvironment (Fig. 1J). Moreover, the expression of ANGPTL4 was correlated with infiltration of multiple immune cell types, including neutrophils, macrophages, mast cells, immature dendritic cells (iDCs), T-helper 1 (Th1) cells, and Tgd (Fig. 1K). Moreover, we confirmed the expression of ANGPTL4 in multiple OC cell lines based on the CCLE database (Fig. 1L), and we chose the Hey-A8 (with low expression of ANGPTL4, moderately differentiated papillary cystadenocarcinoma) and SKOV3 (with high expression of ANGPTL4, moderately well differentiated adenocarcinoma) cell lines for further experimental validation. We further confirmed the expression of ANGPTL4 and ESM1 in immortalized ovarian epithelial cells (HOSE) and multiple OC cell lines by Western blot, including CAOV3, A2780, SKOV3 and Hey-A8 (Fig. 1M).

**Table 1:** The expression of ANGPTL4 in ovarian cancer based on HPA database

Groups	Cases (n)	Low (n)	High (n)	χ^2^	P
Normal tissue	3	3	0	8.775	
Ovarian mucinous cystadenoma	10	1	9		0.0031

**Table 2:** The expression of ANGPTL4 in benign and malignant ovarian diseases based on IHC staining

Groups	Cases (n)	Low (n)	High (n)	χ2	*P*
Normal tissue	20	18	2	51.14	
Ovarian mucinous cystadenoma	20	14	6		0.1138
Teratoma	15	12	3		0.4028
Tissues adjacent to epithelial ovarian carcinoma	35	24	11		0.072
Epithelial ovarian carcinoma	35	6	29		< 0.0001
Tissues adjacent to Ovarian granular cell carcinoma	20	9	11		0.0024
Ovarian granular cell carcinoma	20	3	17		< 0.0001

**Table 3:** The expression of ANGPTL4 in ovarian cancer based on TCGA database

characteristics	Low expression of ANGPTL4	High expression of ANGPTL4	p value
n	190	191	
Clinical stage, n (%)			0.4442
Stage I and Stage II	9 (2.4%)	15 (4%)	
Stage III	150 (39.7%)	146 (38.6%)	
Stage IV	30 (7.9%)	28 (7.4%)	
Tumor status, n (%)			0.2177
Tumor free	40 (11.8%)	32 (9.5%)	
With tumor	126 (37.3%)	140 (41.4%)	
Histologic grade, n (%)			0.5159
G1&G2	25 (6.7%)	21 (5.7%)	
G4&G3	160 (43.1%)	165 (44.5%)	
Venous invasion, n (%)			0.5253
No	25 (23.8%)	16 (15.2%)	
Yes	35 (33.3%)	29 (27.6%)	
Lymphatic invasion, n (%)			0.9361
No	26 (17.4%)	22 (14.8%)	
Yes	54 (36.2%)	47 (31.5%)	
ESM1, n (%)			0.0018
Low	110 (28.9%)	80 (21%)	
High	80 (21%)	111 (29.1%)	
VEGFA, n (%)			2.29E-07
Low	120 (31.5%)	70 (18.4%)	
High	70 (18.4%)	121 (31.8%)	

Moreover, we overexpressed ANGPTL4 in Hey-A8 cell (Additional file 1: Fig. S4A). The downstream differentially expressed genes (DEGs) of ANGPTL4 OE were confirmed by RNA-sequence (Additional file 1: Fig. S4B,  C). The potential functions of 14 downstream DEGs were analyzed by GSVA analysis, which indicated that most of these genes are enriched in the JAK-STAT signaling pathway (Additional file 1: Fig. S4D). KEGG enrichment showed that 14 DEGs were enriched in multiple molecular functions, including cytosolic ribosome, structural constituent of ribosome, Ribosome, and receptor antagonist activity (Additional file 1: Fig. S4E).

#### ANGPTL4 enhances the proliferation and angiogenesis ability of OC cells by activating the JAK pathway in vitro and in vivo

**Fig. 2:**
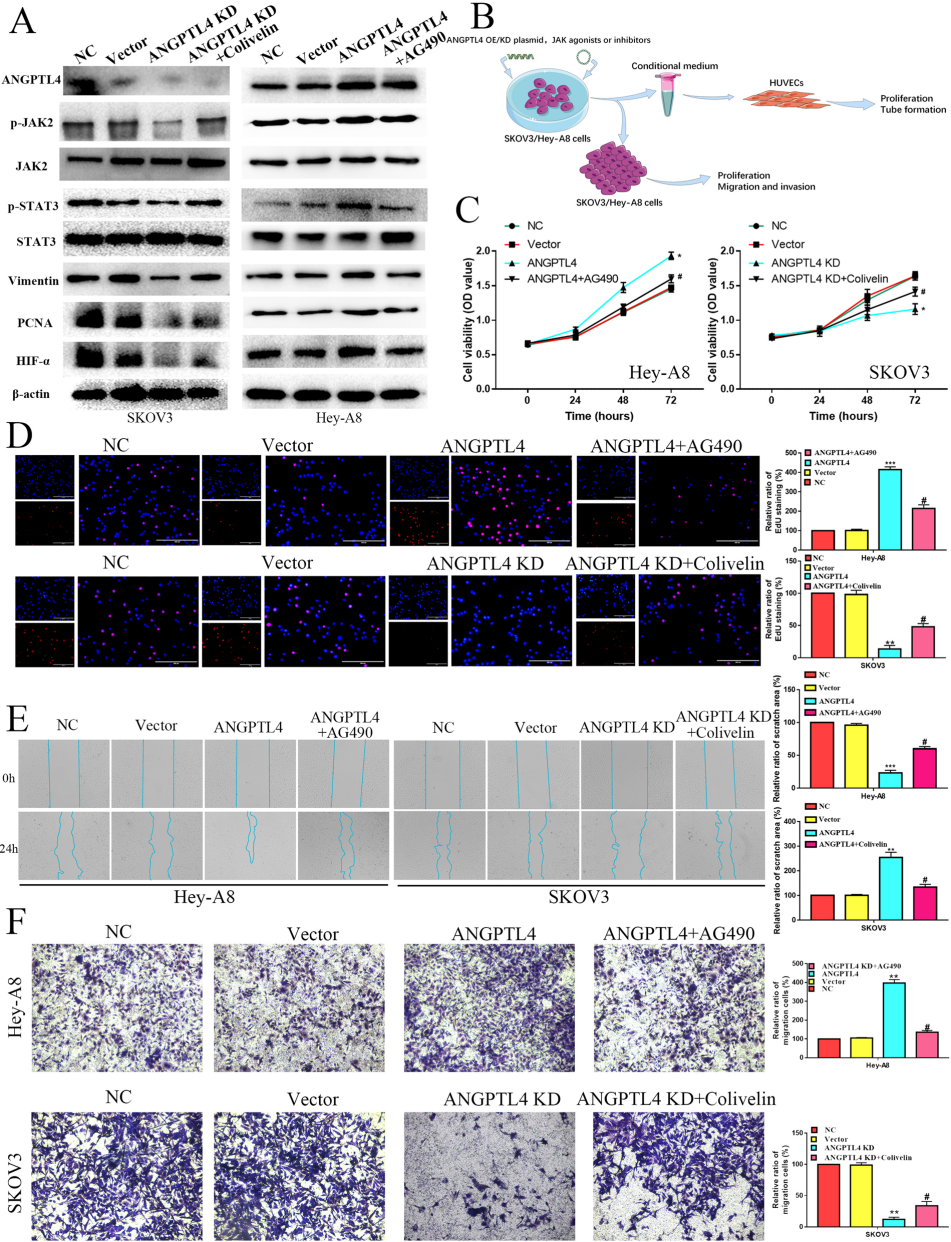
The effects of the ANGPTL4/JAK2/STAT3 pathway on OC proliferation, migration and invasion. **A** The protein levels of ANGPTL4, p-JAK2, JAK2, p-STAT3, STAT3, Vimentin, PCNA and hypoxia-inducible factor alpha (HIF-α) were detected in the NC-SKOV3, Vector-SKOV3, ANGPTL4 KD-SKOV3, ANGPTL4 KD-SKOV3 with Colivelin, NC-HeyA8, Vector-HeyA8, ANGPTL4 OE-HeyA8, and ANGPTL4 OE-HeyA8 with AG490 groups by Western blotting. **B** A workflow for transfecting cells, collecting conditioned medium, and conducting analyses on OC cells and HUVECs. The effect of ANGPTL4, ANGPTL4 + AG490, ANGPTL4 KD, and ANGPTL4 KD + Colivelin on Hey-A8 and SKOV3 cell proliferation ability via MTT (**C**) and EdU (**D**) analysis. The migration and invasion abilities of ANGPTL4, ANGPTL4 + AG490, ANGPTL4 KD, and ANGPTL4 KD + Colivelin were measured by wound healing (**E**) and Transwell invasion assays (**F**) in Hey-A8 and SKOV3 cells, respectively. *P < 0.05, **P < 0.01, ***P < 0.001

**Fig. 3:**
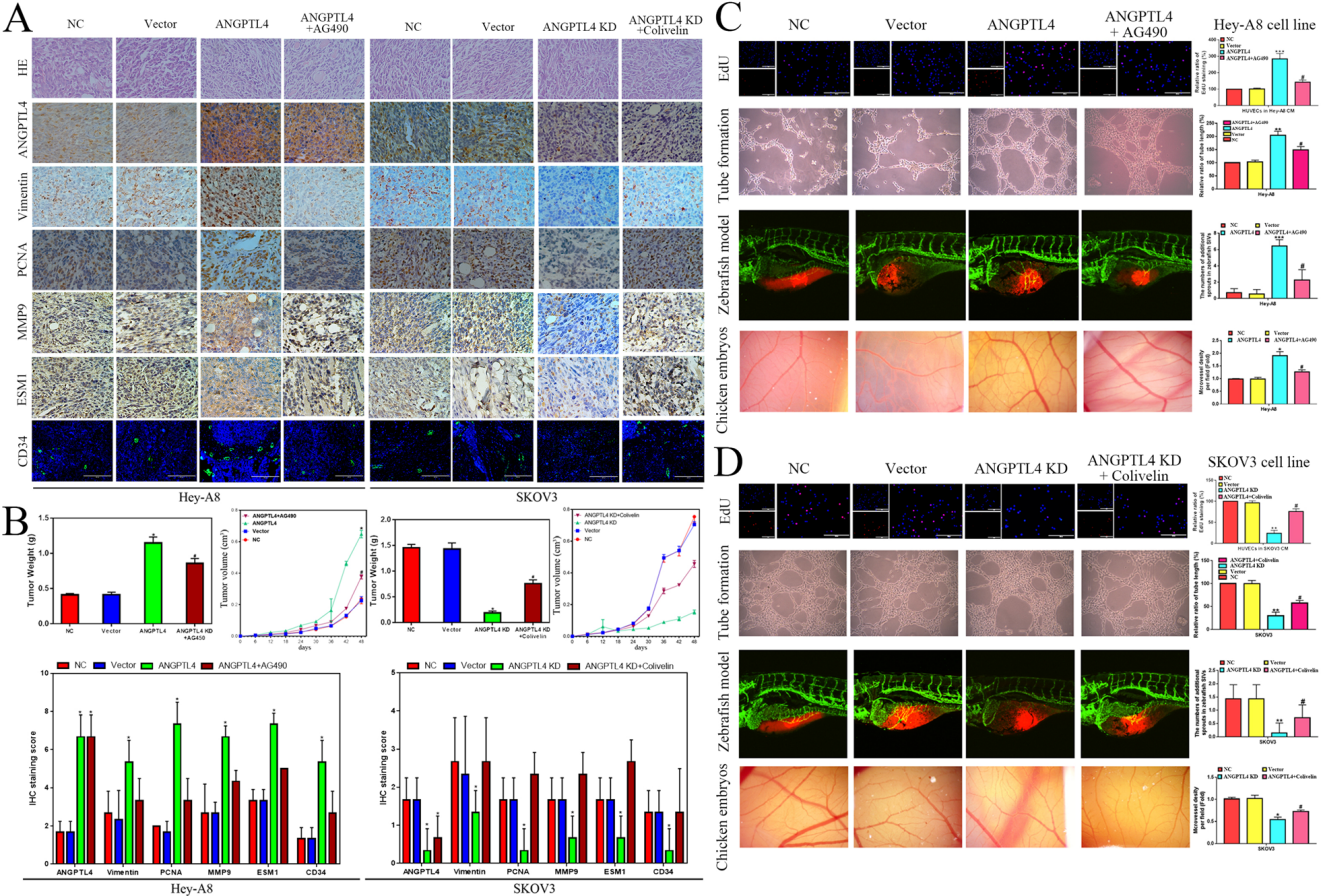
ANGPTL4 promotes OC growth and angiogenesis by activating the JAK2/STAT3 pathway in vivo. **A** Morphological observation and HE and IHC staining of xenografts in the NC-SKOV3, Vector-SKOV3, ANGPTL4 KD-SKOV3, ANGPTL4 KD-SKOV3 with Colivelin, NC-HeyA8, Vector-HeyA8, ANGPTL4 OE-HeyA8, and ANGPTL4 OE-HeyA8 with AG490 groups. **B** The tumor weight and volume and the IHC staining score of xenografts in the NC-SKOV3, Vector-SKOV3, ANGPTL4 KD-SKOV3, ANGPTL4 KD-SKOV3 with Colivelin, NC-HeyA8, Vector-HeyA8, ANGPTL4 OE-HeyA8, and ANGPTL4 OE-HeyA8 with AG490 groups. **C** The proliferation and tube formation of HUVECs cultured with the CM of HeyA8 cells were tested by EdU staining and tube formation assays. The chick chorioallantoic membrane assay measured the angiogenesis ability of HeyA8 CM in vivo. Angiogenesis ability of HeyA8 cells in a zebrafish model. The experimental groups were NC, Vector, ANGPTL4 OE and ANGPTL4 + AG490. **D** The proliferation and tube formation of HUVECs cultured with the CM of SKOV3 cells were tested by EdU staining and tube formation assays. The chick chorioallantoic membrane assay measured the angiogenesis ability of SKOV3 CM in vivo. Angiogenesis ability of SKOV3 cells in the zebrafish model. The experimental groups were NC, Vector, ANGPTL4 KD and ANGPTL4 KD + Colivelin. *P < 0.05, **P < 0.01, ***P < 0.001

According to the GSVA analysis (Additional file 1: Fig. S4D), we constructed ANGPTL4 knockdown or overexpression with JAK-STAT activation or inhibition cell lines, respectively. Western blotting indicated that ANGPTL4 activated the JAK-STAT3 pathway in Hey-A8 and SKOV3 cell lines (Fig. 2A). ELISA analysis was used to detect the level of free ANGPTL4 in the conditioned medium (CM) of each group (Additional file 1: Fig. S5). Moreover, we utilized CM derived from Hey-A8 and SKOV3 cells after ANGPTL4 overexpression/knockdown transfection with JAK inhibitor/activator to incubate human umbilical vein endothelial cells (HUVECs) for six hours, which were used for further proliferation and tube formation analysis (Fig. 2B). MTT analysis indicated that ANGPTL4 overexpression could significantly enhance the proliferation ability of Hey-A8 cells, but the effects of ANGPTL4 overexpression could be rescued by the JAK inhibitor AG490. Conversely, inhibiting the endogenous level of ANGPTL4 by shRNA significantly repressed the proliferation ability of SKOV3 cells in comparison with the vector and normal cell (NC) groups, which could also be rescued by the JAK activator Colivelin (Fig. 2C). Further EdU analysis showed that the proliferation ability of Hey-A8 cells with ANGPTL4 overexpression was obviously and significantly greater than that of vector and NC cells, which could also be rescued by AG490. Conversely, the proliferation ability of SKOV3 cells was significantly reduced upon endogenous ANGPTL4 knockdown, and the effects could also be rescued by Colivelin (Fig. 2D). Wound-healing assays showed that ANGPTL4 overexpression or knockdown could significantly increase or decrease the migration ability of Hey-A8 or SKOV3 cells, respectively, which could also be rescued by a JAK inhibitor or activator, respectively (Fig. 2E). Transwell invasion assays indicated that the invasion ability of Hey-A8 cells with ANGPTL4 overexpression was significantly enhanced compared to that of vector cells, but SKOV3 with ANGPTL4 knockdown cell invasion ability was remarkedly reduced in comparison with that of the vector and NC groups. These effects could be rescued by a JAK inhibitor or activator (Fig. 2F). To study the effects of ANGPTL4 on tumor growth in vivo, a xenograft mouse model was used. ANGPTL4 overexpression could promote OC cell growth (tumor weight and volume) with higher expression of vimentin, proliferating cell nuclear antigen (PCNA), MMP9, ESM1 and CD34, which could be rescued by AG490. Conversely, ANGPTL4 inhibition significantly repressed the tumor weight and volume of SKOV3 cells with low vimentin, PCNA, MMP9, ESM1 and CD34 expression in comparison with the vector and NC groups, which could also be rescued by Colivelin (Fig. 3A, B). To further confirm the role of ANGPTL4 in OC angiogenesis, we found that ANGPTL4 overexpression could significantly promote HUVECs cultured with Hey-A8 cell CM proliferation (by EdU analysis) and angiogenesis (by tube formation) in vitro and in vivo (by zebrafish model and chicken embryos), which could be rescued by AG490 (Fig. 3C). Similarly, angiogenesis was obviously retarded in the CM of SKOV3 cells with ANGPTL4 knockdown in vitro and in vivo and rescued by a JAK activator (Fig. 3D). Moreover, we confirmed the expression of ESM1 was enhanced by JAK activator Colivelin and reduced by JAK inhibitor AG490 (Additional file 1: Fig. S6A). The angiogenesis effect of JAK-STAT pathway was dependent on the ESM1 expression in OC (Additional file 1: Fig. S6B). WB showed that ESM1 knockdown could repress ANGPTL4 expression, and ANGPTL4 knockdown could also reduce the expression of ESM1, which indicated that ANGPTL4 and ESM1 could form positive feedback to accelerate OC carcinogenesis (Additional file 1: Fig. S6C). Furthermore, Co-IP analysis showed that JAK activator Colivelin had no significant effect on the binding of ESM1 and ANGPTL4 in SKOV3 cells (Additional file 1: Fig. S6D). Taken together, these results indicated that ANGPTL4 could promote OC cell proliferation, migration, invasion, and angiogenesis by promoting the phosphorylation and activation of JAK-STAT pathway in vitro and in vivo.

### ANGPTL4 physically and functionally interacts with ESM1 in OC cells

**Fig. 4:**
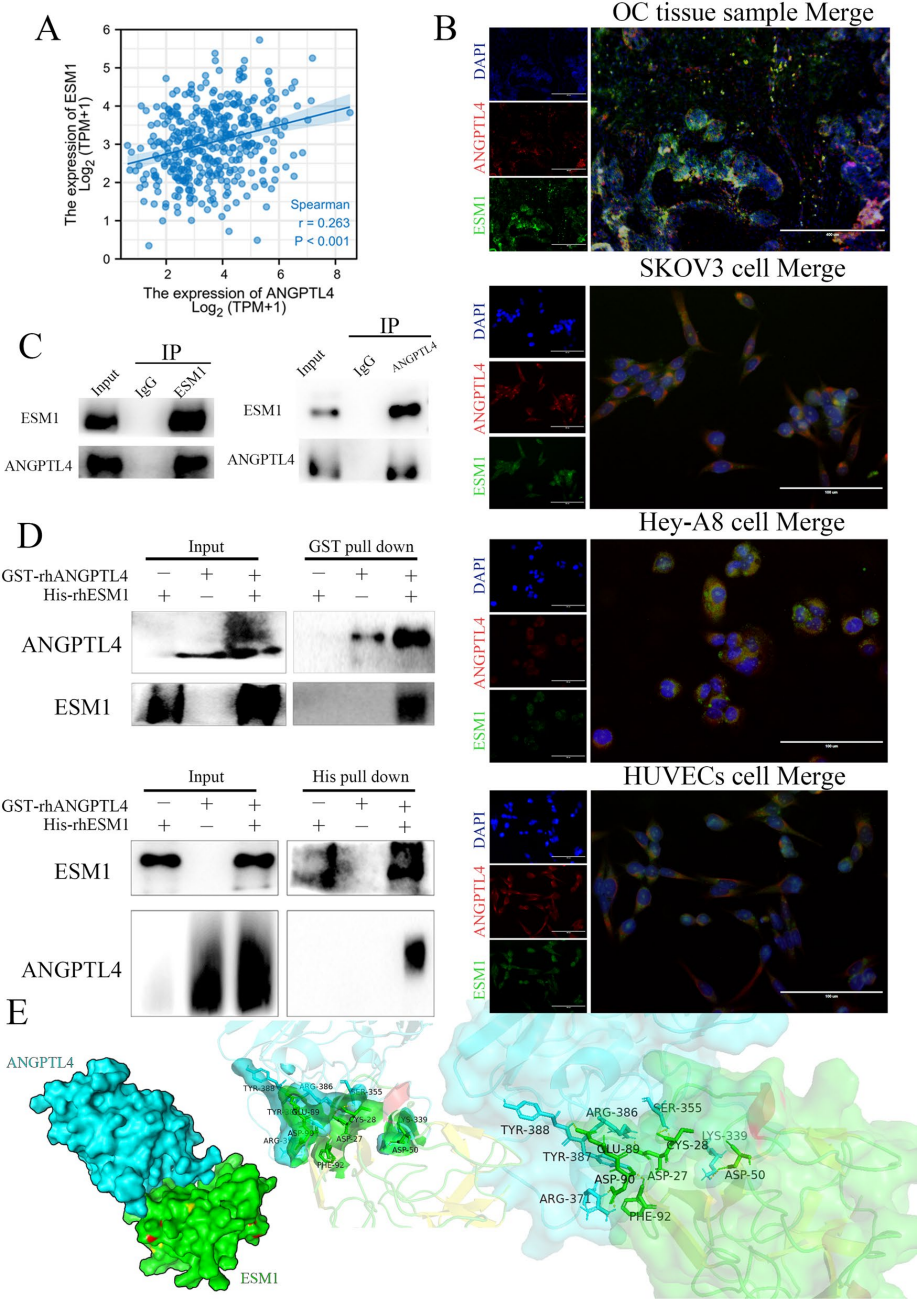
ANGPTL4 interacted with ESM1. **A** The correlation expression of ESM1 and ANGPTL4 in OC samples based on TCGA database. **B** IF staining for ANGPTL4 and ESM1 in OC tissue samples, SKOV3 cell lines, HeyA8 cell lines and HUVECs. **C** The interaction of ANGPTL4 and ESM1 in HeyA8 cells by Co-IP analysis. **D** GST pull-down analysis for GST-ANGPTL4 and His-ESM1. **E** Molecular docking for ANGPTL4 and ESM1 by HADDOCK.

ANGPTL4 plays a key role in molecular biology processes, such as an unfolding molecular chaperone [27, 28]. ESM1 is characterized by interacting with the armadillo repeat domain (ARM) of β-catenin to activate the Wnt pathway [16]. We wondered whether there are potential regulatory mechanisms between ANGPTL4 and ESM1. Coexpression analysis showed that ANGPTL4 was significantly and positively correlated with ESM1 in OC patients based on the TCGA database (Fig. 4A). In our IF staining, colocalization was observed between ESM1 and ANGPTL4 staining in OC samples (Fig. 4B). IF analysis revealed a high degree of colocalization between ANGPTL4 and ESM1 in the cytoplasm of OC cells and endothelial cells (Fig. 4B). Indeed, the presence of ESM1 was detected in the immune complex precipitated by the anti-ANGPTL4 antibody from either the nuclear or cytoplasmic fraction of SKOV3 cells (Fig. 4C). The direct binding of His-recombinant human ESM1 (rhESM1) and GST-tagged ANGPTL4 protein was confirmed in reciprocal pull-down assays (Fig. 4D). We next docked the ESM1 protein, created by homology modeling, with the ANGPTL4 protein from the PDB database (6U1U) using HADDOCK (High Ambiguity-Driven biomolecular docking). ESM1 was predicted to bind near the long positively charged groove constituted by the C-terminal fibrinogen-like domain in the molecular docking model (Fig. 4E). By PISA (protein interfaces, structures, and assemblies) analysis, the thermodynamic stability of the ESM1-ANGPTL4 complex was estimated to predict possible interaction residues and calculate binding surface areas. Three residues, Arg-371, Tyr-387, Tyr-388, Arg-386, Ser-355 and Lys-339, in ANGPTL4 were predicted to be crucial in the binding region, which indicated that the interaction between ESM1 and ANGPTL4 might be a crucial function in the molecular functions of ANGPTL4.

#### The ANGPTL4/ESM1 interaction promotes OC carcinogenesis in vivo by inhibiting lipoprotein lipase (LPL) activity

**Fig. 5:**
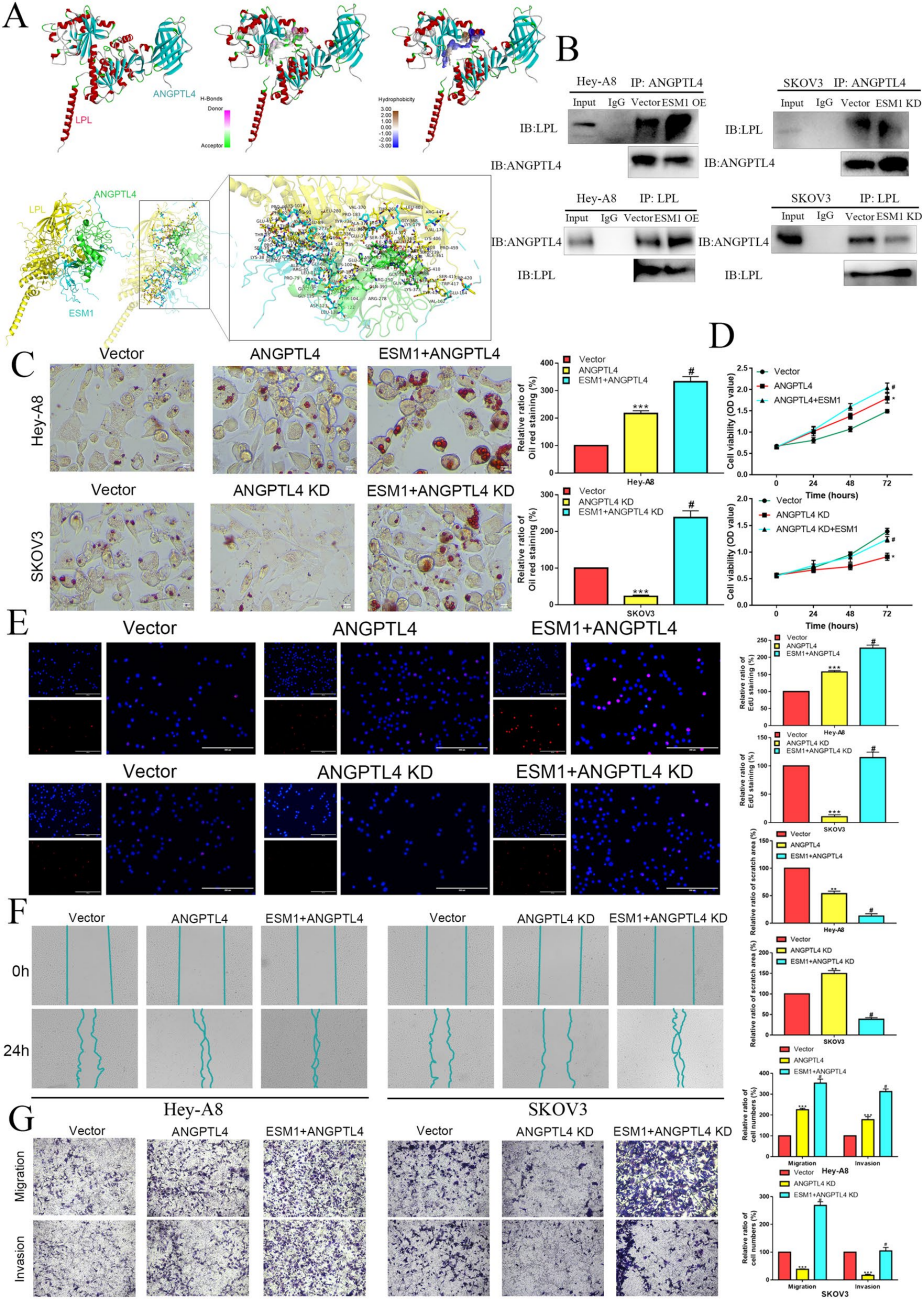
ESM1 promotes ANGPTL4 to combine with LPL by accelerating proliferation, invasion and lipid accumulation in OC cells. **A** Molecular docking for ANGPTL4, LPL and ESM1 by HADDOCK. **B** Co-IP showed the effects of ESM1 on the interaction between ANGPTL4 and LPL in HeyA8 and SKOV3 cells. The effect of vector, ANGPTL4, ANGPTL4 + ESM1, ANGPTL4 KD, and ANGPTL4 KD + ESM1 on Hey-A8 and SKOV3 cell lipid levels and proliferation ability via oil red O staining (**C**), MTT (**D**) and EdU (**E**) analysis. The migration and invasion abilities were measured by wound healing (**F**) and Transwell assays (**G**) in Hey-A8 and SKOV3 cells transfected with vector, ANGPTL4, ANGPTL4 + ESM1, ANGPTL4 KD, and ANGPTL4 KD + ESM1. *P < 0.05, **P < 0.01, ***P < 0.001

In a previous study, ANGPTL4 bound to LPL sequences adjacent to the catalytic cavity, triggering sequential and cooperative unfolding of the hydrolase domain for LPL, which induced irreversible destruction of the catalytic cavity and LPL inactivation [29]. We next examined the effects of the ANGPTL4/ESM1 interaction on LPL activity. As expected, our docking model indicated that they may not compete with each other. As shown in Fig. 5A, ESM1 binds to ANGPTL4, while LPL binds to ANGPTL4. To confirm whether ESM1 promotes ANGPTL4 binding to LPL, coimmunoprecipitation indicated that the interaction of ANGPTL4 and LPL was increased after ESM1 overexpression in Hey-A8 cells (Fig. 5B). Conversely, the interaction between ANGPTL4 and LPL was decreased when ESM1 was knocked down in SKOV3 cells (Fig. 5B).

Furthermore, we used oil red O staining to confirm the effects of the ANGPTL4/ESM1 interaction on OC cells. ANGPTL4 overexpression obviously enhanced the lipid level in Hey-A8 cells, which was consolidated by ESM1 overexpression. SKOV3 cells with ANGPTL4 knockdown had lower lipid levels than vector cells, and ESM1 overexpression rescued these molecular functions (Fig. 5C). MTT analysis showed that ANGPTL4 overexpression could promote the viability of Hey-A8 cells, and ESM1 could synergistically promote cell viability with ANGPTL4 in Hey-A8 cells. Similarly, ANGPTL4 knockdown inhibited the proliferation of SKOV3 cells, which was rescued by ESM1 overexpression (Fig. 5D). EdU analysis also indicated that ANGPTL4 could promote OC cell proliferation, and ESM1 could consolidate the molecular functions of ANGPTL4 for promoting cell proliferation (Fig. 5E). Wound-healing assays showed that ANGPTL4 overexpression or knockdown could significantly increase or decrease the migration ability of Hey-A8 or SKOV3 cells, respectively, which could also be amplified or rescued by ESM1 overexpression, respectively (Fig. 5F). Transwell invasion assays indicated that the invasion ability of Hey-A8 cells with ANGPTL4 overexpression was significantly enhanced compared to that of vector cells, but SKOV3 cells with ANGPTL4 knockdown had markedly reduced invasion ability in comparison with that of the vector group. These effects could be amplified or rescued by ESM1 overexpression (Fig. 5G).

Then, we found that ANGPTL4 overexpression could promote xenograft growth with higher expression of vimentin, PCNA and CD34, and ESM1 could consolidate the effect of ANGPTL4 on Hey-A8 growth in vivo. Similarly, ANGPTL4 knockdown inhibited the xenograft growth of SKOV3 cells with low expression of vimentin, PCNA and CD34, which was rescued by ESM1 overexpression in vivo (Additional file 1: Fig. S7A, B).

Next, we found that ANGPTL4 could interact with ESM1 to promote tube formation in vitro and accelerate vascularization in vivo for Hey-A8 cells (Additional file 1: Fig. S7C). ANGPTL4 knockdown suppressed the angiogenesis ability of SKOV3 cells, but ESM1 overexpression rescued the molecular effects of ANGPTL4 knockdown on OC cells in vitro and in vivo (Additional file 1: Fig. S7D). Overall, ESM1 could cooperate with ANGPTL4 to accelerate OC lipid metabolism, proliferation and angiogenesis in vitro and in vivo.

#### Interaction of ANGPTL4 with ESM1 modulates vascular junction integrity to disrupt integrin α5β1 and intercellular VE-Cad in endothelial cells in the OC tumor microenvironment

**Fig. 6:**
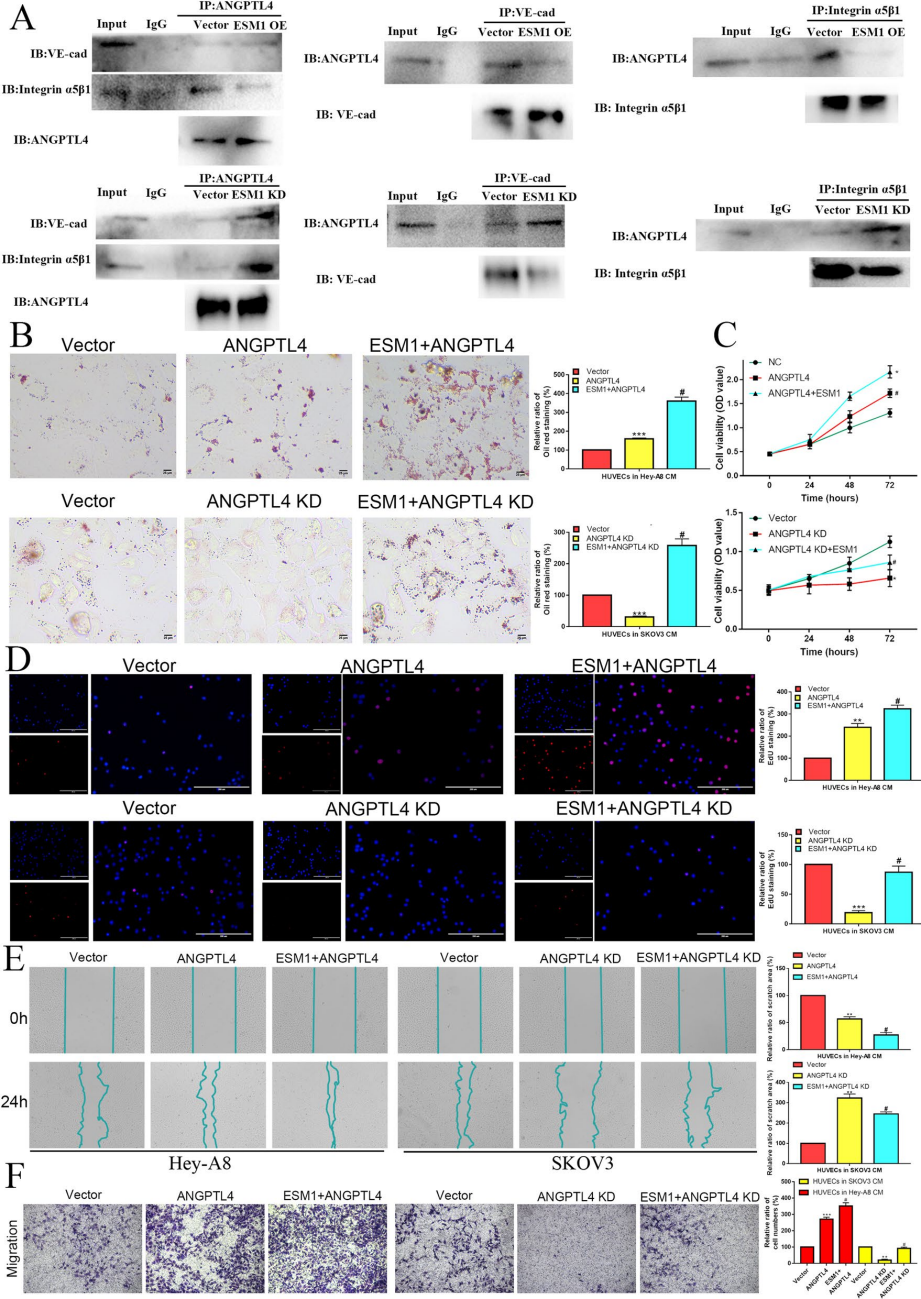
ESM1 inhibits ANGPTL4 binding to integrin α5β1 and VE-cadherin, which represses HUVEC proliferation and migration to induce vascular permeability in the tumor microenvironment. **A** Co-IP showed that ESM1 inhibited ANGPTL4 binding to α5β1 and VE-cadherin in HUVECs cultured with HeyA8 and SKOV3 cells, respectively. The effect of vector, ANGPTL4, ANGPTL4 + ESM1, ANGPTL4 KD, and ANGPTL4 KD + ESM1 on HUVECs cultured with Hey-A8 and SKOV3 cell lipid levels and proliferation ability via oil red O staining (**B**), MTT (**C**) and EdU (**D**) analysis. The migration ability was measured by wound healing (**F**) and Transwell assays (**G**) in HUVECs cultured with Hey-A8 and SKOV3 cells transfected with vector, ANGPTL4, ANGPTL4 + ESM1, ANGPTL4 KD, and ANGPTL4 KD + ESM1. *P < 0.05, **P < 0.01, ***P < 0.001

Integrin α5β1 and VE-Cad play important molecular biological roles in neovascularization, so we further investigated the key role of ANGPTL4-ESM1 interactions in these systems. Our coimmunoprecipitation analysis showed that VE-Cad and integrin α5β1 binding to ANGPTL4 was significantly decreased in HUVECs cultured with CM from Hey-A8 cells overexpressing ESM1, but VE-Cad and integrin α5β1 binding to ANGPTL4 was obviously increased in HUVECs cultured with CM from SKOV3 cells with ESM1 knockdown (Fig. 6A). The interaction of ANGPTL4 with VE-Cad or integrin α5β1 might attenuate the connection and adhesion between endothelial cells [6]. Therefore, we further examined the role of the ESM1/ANGPTL4 interaction on the molecular biological behavior of endothelial cells in the OC tumor microenvironment. Our results showed that ANGPTL4 overexpression could significantly promote lipid accumulation (by oil red O staining), proliferation (by EdU analysis), and migration (by wound healing and transwell migration assays) in vitro, which could be collaboratively intensified by ESM1 overexpression in HUVECs cultured with Hey-A8 cell CM (Fig. 6B***–***F). Similarly, the lipid level, proliferation and migration ability were obviously enhanced in HUVECs cultured with CM from SKOV3 cells with ANGPTL4 knockdown and rescued by ESM1 overexpression (Fig. 6B***–***F). Taken together, these results indicated that ESM1 could inhibit ANGPTL4 binding to integrin α5β1 and VE-Cad, which enhanced the proliferation and migration of HUVECs and delayed vascular disruption to induce angiogenesis.

**Fig. 7:**
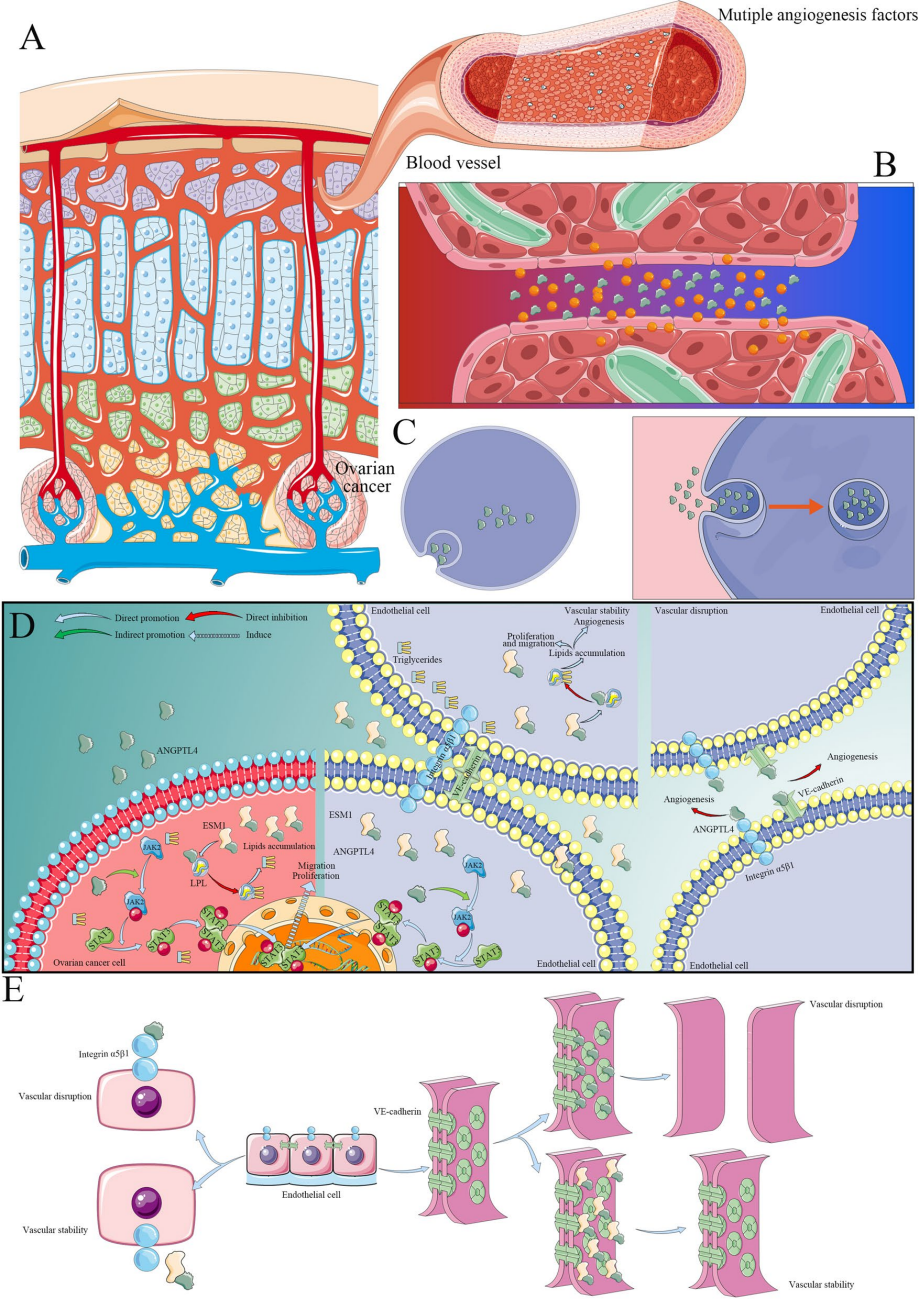
A model of the molecular mechanism by which ANGPTL4 promotes OC development and progression. **A** OC is a type of tumor with a rich blood supply. **B** There are many angiogenic factors in the tumor microenvironment of OC. **C** A variety of cells in the tumor microenvironment can take up these angiogenesis factors in a variety of ways to produce different pathophysiological effects. **D** ANGPTL4 can promote the proliferation and migration of OC cells by activating the JAK-STAT3 pathway, and ESM1 can bind ANGPTL4 and stabilize the binding of ANGPTL4 to LPL, thus promoting lipid accumulation and accelerating the proliferation and migration of OC cells. ANGPTL4 can also promote endothelial cell proliferation and migration through the JAK-STAT3 signaling pathway. ESM1 can inhibit the binding of ANGPTL4 to integrin α5β1 to increase vascular stability. Moreover, ESM1 promotes angiogenesis in the ovarian cancer microenvironment by binding to ANGPTL4 to inhibit its destruction of VE-cadherin-mediated tight junctions. **E** The molecular mechanism might be attributed to the interaction of ESM1 and ANGPTL4.

## Discussion

In this study, we determined that ANGPTL4 was an important factor with multiple molecular biological functions, prognostic significance and ectopic expression among ARGs in OC progression. IHC staining analysis indicated that ANGPTL4 was obviously increased in OC patients at the protein level. Multiple studies have indicated that ANGPTL4 is a functionally complex protein in many types of cancer, including gastric cancer [13, 30], OC [31], colon cancer [32], and breast cancer [33]. Our RNA sequencing results showed that ANGPTL4 might activate the JAK2-STAT3 pathway by regulating downstream gene expression in OC. Chong et al. also found that ANGPTL4 could activate STAT3/ Inducible nitric oxide synthase (iNOS) to upregulate angiogenesis, resulting in the promotion of wound healing in mice with diabetes [34]. Avalle and his colleagues found that STAT3 could promote the secretion of ANGPTL4 to accelerate cell growth in breast cancer [9]. ANGPTL4 promoted OC cell proliferation, migration, invasion and angiogenesis by activating the JAK2/STAT3 signaling pathway in vitro and in vivo.

In our previous study, we found that ESM1 was a key secretory protein that promoted OC cell proliferation, apoptosis escape, migration, invasion and angiogenesis [20]. The functions of ESM1 and ANGPTL4 overlap in OC development and progression. We found that ANGPTL4 expression was significantly correlated with ESM1 in OC samples, OC and endothelial cells. Moreover, Co-IP and GST pull-down analyses showed that ESM1 could bind to ANGPTL4, indicating a direct interaction. ANGPTL4 can interact with LPL to attenuate lipidolysis [35]. Our molecular docking and Co-IP analysis indicated that ESM1 stabilizes the interaction of ANGPTL4 and LPL. In a previous study, Baczewska et al. found that LPL expression was decreased and correlated with favorable prognosis in OC patients [36]. Metabolic reprogramming is a characteristic feature of metastatic OC cells in malignant ascites. In contrast to primary OC tumors, a large number of cytoplasmic lipid droplets and lipid phagocytic vesicles accumulate in OC metastatic cells [37]. LPL can hydrolyze triglycerides in follicular tissues to maintain normal lipid homeostasis [38]. ANGPTL4 mediates the inhibition of LPL activity by promoting the unfolding of the LPL protein domain, leading to the cleavage and degradation of LPL [39]. In our study, we found that ESM1 could promote.

The interaction between ANGPTL4 and LPL upregulated lipid levels, resulting in lipid metabolism reprogramming in OC cells. The alteration of lipid metabolism will further induce the growth and migration of cancer cells [40, 41]. We also found that lipid accumulation mediated by ESM1 and ANGPTL4 could promote OC cell proliferation and invasion in vivo and in vitro.

Moreover, the combined effect of ESM1 and ANGPTL4 on lipid accumulation, cell proliferation and migration was also observed in endothelial cells cultured from the CM of OC cells. These molecular functions of ESM1/ANGPTL4 significantly promote angiogenesis in the OC tumor microenvironment. However, ANGPTL4 has completely different molecular biological functions in different diseases and even in different cell lines of the same disease. Under physiological conditions, ANGPTL4 can inhibit the motility of endothelial cells and attenuate sprouting and tube formation to repress angiogenesis by possibly inactivating the MAPK pathway in endothelial cells [42]. In cancer studies, the effect of ANGPTL4 on angiogenesis appears to be dual and contradictory, especially in gastric cancer [13, 43, 44] and OC [31, 45, 46]. We found that ANGPTL4 could accelerate OC proliferation, migration, invasion and angiogenesis in vivo and in vitro, possibly attributed to activating the JAK-STAT pathway and interacting with ESM1. Rac Family Small GTPase (Rac-GTP) and P21 Activated Kinase (PAK) serve as downstream effectors of integrin signaling, whose pivotal roles in regulating endothelial contractility and barrier function have been well established [47, 48]. Furthermore, we found that ESM1 could attenuate ANGPTL4 interaction with integrin α5β1 and VE-Cad, resulting in the maintenance of tight junctions and vascular stability. Royston-Luke Huang et al. also indicated that cANGPTL4 disrupts endothelial continuity by interacting with integrin α5β1 and VE-Cad [6]. The interaction of ANGPTL4 with different proteins in the tumor microenvironment may be one of the reasons for its paradoxical effect on angiogenesis in different diseases or cell lines.

Overall, this study indicated that ANGPTL4 promotes OC cell proliferation, migration, invasion, and angiogenesis by activating the JAK-STAT pathway in vivo and in vitro. Moreover, the interaction of ANGPTL4 and ESM1 drives lipid metabolism reprogramming to promote OC cell proliferation and invasion by inactivating LPL. This interaction also accelerates the proliferation, migration, and vascular junction integrity of endothelial cells to promote angiogenesis by stabilizing integrin α5β1 and VE-Cad in the tumor microenvironment (Fig. 7).

### Supplementary Information

**Additional file 1:  Fig. S1.** Prognostic significance of ARGs in OC patients. **A** Prognostic models were constructed by LASSO regression based on ARGs. **B** Lambda on abscissa, coefficients on ordinate. **C** The survival times, risk score, and signature expression in OC patients. **D** Overall survival analysis for OC patients with high or low risk. **E** ROC curves for survival analysis. **F** Correlation analysis between the risk score and immune cell infiltration. *P < 0.05, **P < 0.01, ***P < 0.001. **Fig. S2.** Hub prognostic ARGs in OC.  Uni_cox (**A**) and Mult_cox (**B**) for 13 signatures in OC. Overall survival significance confirmed by the nomogram (**C**) and calibration curve (**D**). Drug sensitivity analysis for EGF, ANGPTL4, RUNX1, PLG, and NOTCH4 in the CTRP database (**E**) and GDSC database (**F**). PCR analysis (**G**) for the level of ANGPTL4 and ESM1 in SKOV3 treated with different doses of bevacizumab *P < 0.05, **P < 0.01, ***P < 0.001. **Fig. S3.** The expression of ANGPTL4 in pan-carcinoma based TCGA database and GTEx database. The blue is for normal tissue samples and the red is for cancer samples.  **Fig. S4.** The expression of downstream genes in HeyA8 cells after ANGPTL4 overexpression.  **A**  ANGPTL4 expression confirmed in Hey-A8 cells by IF staining.  **B**  Volcano plot for DEG expression after ANGPTL4 overexpression by RNA sequencing. **C**  Heatmaps for DEG expression. **D** GSVA analysis and E  KEGG analysis for 14 DEGs.  **Fig. S5.** The level of free ANGPTL4 in CM. Free ANGPTL4 expression confirmed in the CM of SKOV3 and Hey-A8 cells by ELISA. *P < 0.05.  **Fig. S6.** ESM1 was a key factor in the downstream of JAK-STAT pathway.  **A**  The expression of ESM1 in SKOV3-DMSO, SKOV3-Colivelin, Hey-A8-DMSO, and Hey-A8-AG490 groups. **B**  The effect of ESM1 on the angiogenesis ability of OC induced by JAK inhibitor/activator. **C**  The effect of ANGPTL4 on ESM1 expression. **D**  Co-IP showed the effects of JAK activator Colivelin on the interaction between ANGPTL4 and ESM1 in SKOV3 cells. **Fig. S7.** The ANGPTL4/ESM1 axis promotes OC growth and angiogenesis in vivo.  **A**  Morphological observation, HE and IHC staining of xenografts in the vector-SKOV3, ANGPTL4 KD-SKOV3, ANGPTL4 KD-SKOV3 with ESM1 OE, vector-HeyA8, ANGPTL4 OE-HeyA8, and ANGPTL4 OE-HeyA8 with ESM1 OE groups. **B**  The tumor weight and volume and the IHC staining score of xenografts in the vector-SKOV3, ANGPTL4 KD-SKOV3, ANGPTL4 KD-SKOV3 with ESM1 OE, vector-HeyA8, ANGPTL4 OE-HeyA8, and ANGPTL4 OE-HeyA8 with ESM1 OE groups. *P < 0.05.

## Data Availability

All data generated or analyzed during this study are included in this published article (and its Additional information files).
