# Supplementary figures and images for: ANGPTL4 accelerates ovarian serous cystadenocarcinoma carcinogenesis and angiogenesis in the tumor microenvironment by activating the JAK2/STAT3 pathway and interacting with ESM1
Source: J Transl Med. 2024 Jan 11;22:46. doi: 10.1186/s12967-023-04819-8 (PMC10785435; doi:10.1186/s12967-023-04819-8)

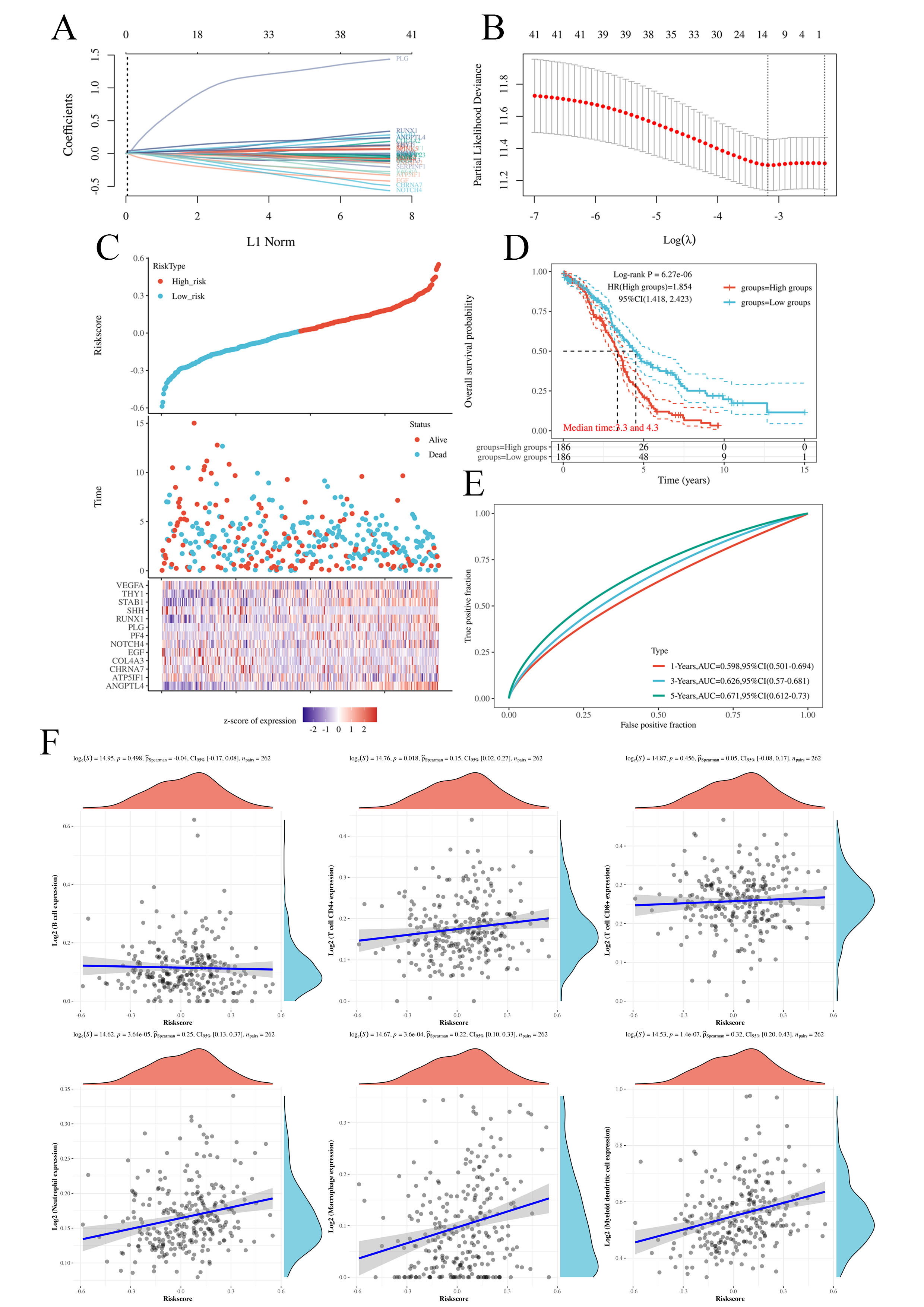

Supplement: Supplementary file 1 — Additional file 1: Fig. S1. Prognostic significance of ARGs in OC patients. A Prognostic models were constructed by LASSO regression based on ARGs. B Lambda on abscissa, coefficients on ordinate. C The survival times, risk score, and signature expression in OC patients. D Overall survival analysis for OC patients with high or low risk. E ROC curves for survival analysis. F Correlation analysis between the risk score and immune cell infiltration. *P < 0.05, **P < 0.01, ***P < 0.001. Fig. S2. Hub prognostic ARGs in OC. Uni_cox (A) and Mult_cox (B) for 13 signatures in OC. Overall survival significance confirmed by the nomogram (C) and calibration curve (D). Drug sensitivity analysis for EGF, ANGPTL4, RUNX1, PLG, and NOTCH4 in the CTRP database (E) and GDSC database (F). PCR analysis (G) for the level of ANGPTL4 and ESM1 in SKOV3 treated with different doses of bevacizumab *P < 0.05, **P < 0.01, ***P < 0.001. Fig. S3. The expression of ANGPTL4 in pan-carcinoma based TCGA database and GTEx database. The blue is for normal tissue samples and the red is for cancer samples. Fig. S4. The expression of downstream genes in HeyA8 cells after ANGPTL4 overexpression. A ANGPTL4 expression confirmed in Hey-A8 cells by IF staining. B Volcano plot for DEG expression after ANGPTL4 overexpression by RNA sequencing. C Heatmaps for DEG expression. D GSVA analysis and E KEGG analysis for 14 DEGs. Fig. S5. The level of free ANGPTL4 in CM. Free ANGPTL4 expression confirmed in the CM of SKOV3 and Hey-A8 cells by ELISA. *P < 0.05. Fig. S6. ESM1 was a key factor in the downstream of JAK-STAT pathway. A The expression of ESM1 in SKOV3-DMSO, SKOV3-Colivelin, Hey-A8-DMSO, and Hey-A8-AG490 groups. B The effect of ESM1 on the angiogenesis ability of OC induced by JAK inhibitor/activator. C The effect of ANGPTL4 on ESM1 expression. D Co-IP showed the effects of JAK activator Colivelin on the interaction between ANGPTL4 and ESM1 in SKOV3 cells. Fig. S7. The ANGPTL4/ESM1 axis promotes OC growth and a [file 12967_2023_4819_MOESM1_ESM.zip › Supplementary/Supplementary Fig1.jpg]

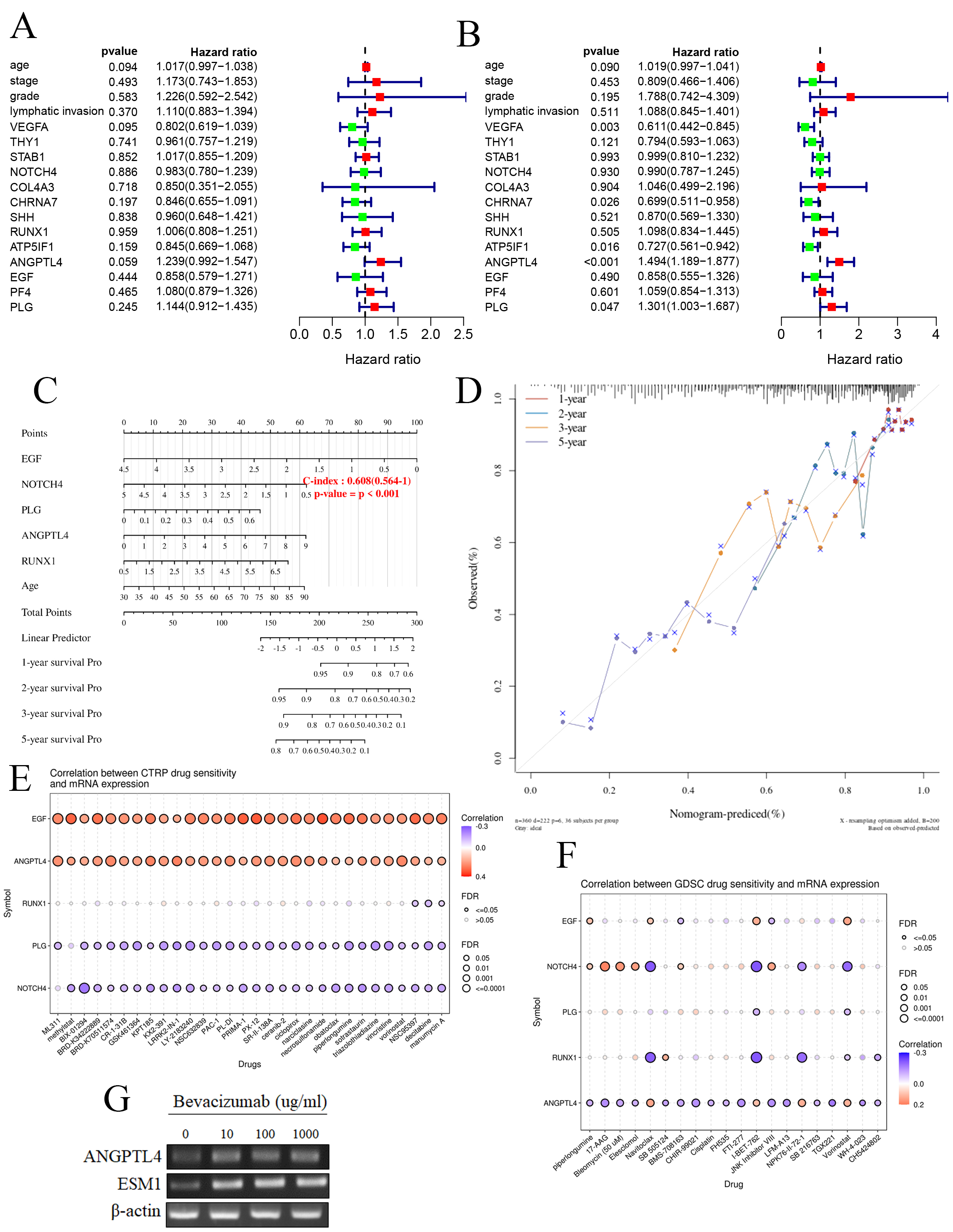

Supplement: Supplementary file 1 — Additional file 1: Fig. S1. Prognostic significance of ARGs in OC patients. A Prognostic models were constructed by LASSO regression based on ARGs. B Lambda on abscissa, coefficients on ordinate. C The survival times, risk score, and signature expression in OC patients. D Overall survival analysis for OC patients with high or low risk. E ROC curves for survival analysis. F Correlation analysis between the risk score and immune cell infiltration. *P < 0.05, **P < 0.01, ***P < 0.001. Fig. S2. Hub prognostic ARGs in OC. Uni_cox (A) and Mult_cox (B) for 13 signatures in OC. Overall survival significance confirmed by the nomogram (C) and calibration curve (D). Drug sensitivity analysis for EGF, ANGPTL4, RUNX1, PLG, and NOTCH4 in the CTRP database (E) and GDSC database (F). PCR analysis (G) for the level of ANGPTL4 and ESM1 in SKOV3 treated with different doses of bevacizumab *P < 0.05, **P < 0.01, ***P < 0.001. Fig. S3. The expression of ANGPTL4 in pan-carcinoma based TCGA database and GTEx database. The blue is for normal tissue samples and the red is for cancer samples. Fig. S4. The expression of downstream genes in HeyA8 cells after ANGPTL4 overexpression. A ANGPTL4 expression confirmed in Hey-A8 cells by IF staining. B Volcano plot for DEG expression after ANGPTL4 overexpression by RNA sequencing. C Heatmaps for DEG expression. D GSVA analysis and E KEGG analysis for 14 DEGs. Fig. S5. The level of free ANGPTL4 in CM. Free ANGPTL4 expression confirmed in the CM of SKOV3 and Hey-A8 cells by ELISA. *P < 0.05. Fig. S6. ESM1 was a key factor in the downstream of JAK-STAT pathway. A The expression of ESM1 in SKOV3-DMSO, SKOV3-Colivelin, Hey-A8-DMSO, and Hey-A8-AG490 groups. B The effect of ESM1 on the angiogenesis ability of OC induced by JAK inhibitor/activator. C The effect of ANGPTL4 on ESM1 expression. D Co-IP showed the effects of JAK activator Colivelin on the interaction between ANGPTL4 and ESM1 in SKOV3 cells. Fig. S7. The ANGPTL4/ESM1 axis promotes OC growth and a [file 12967_2023_4819_MOESM1_ESM.zip › Supplementary/Supplementary Fig2.jpg]

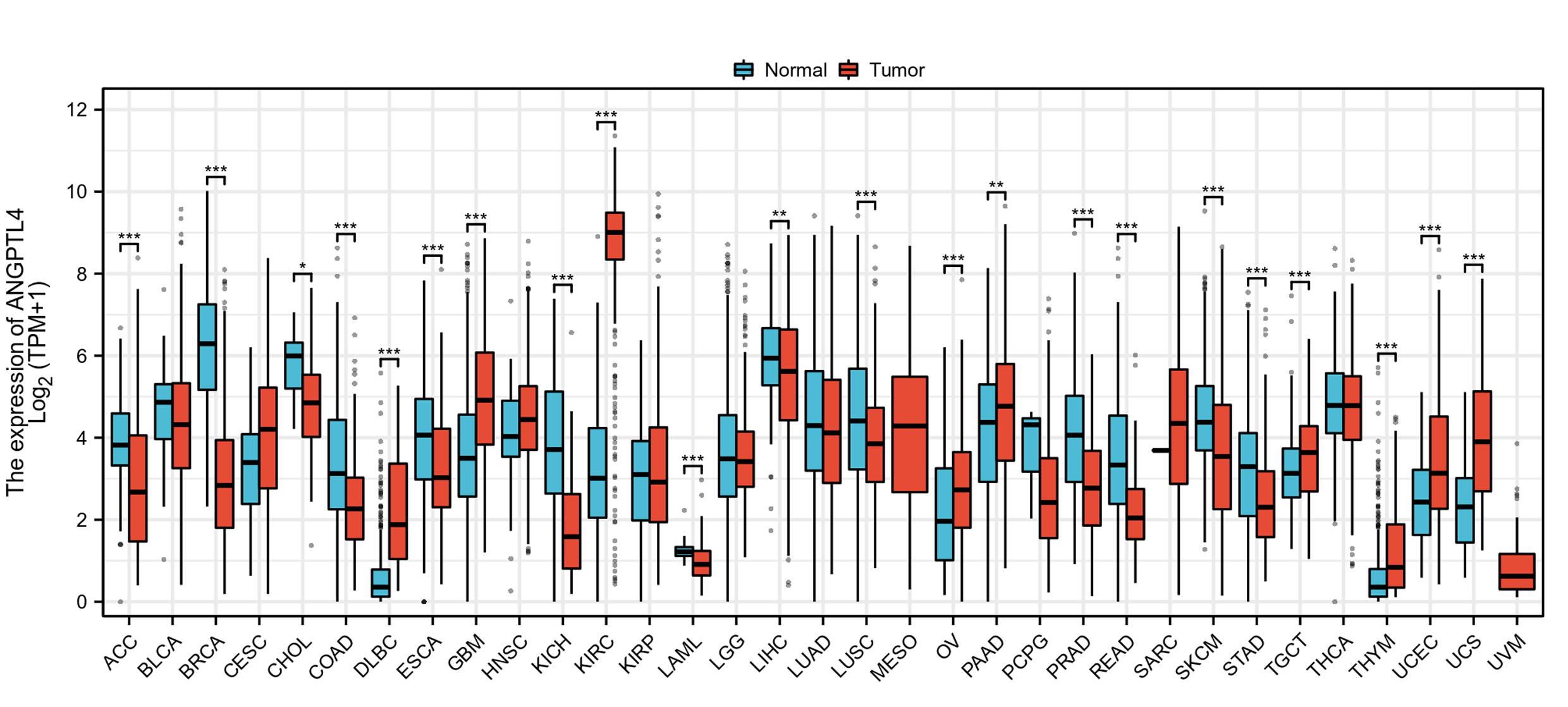

Supplement: Supplementary file 1 — Additional file 1: Fig. S1. Prognostic significance of ARGs in OC patients. A Prognostic models were constructed by LASSO regression based on ARGs. B Lambda on abscissa, coefficients on ordinate. C The survival times, risk score, and signature expression in OC patients. D Overall survival analysis for OC patients with high or low risk. E ROC curves for survival analysis. F Correlation analysis between the risk score and immune cell infiltration. *P < 0.05, **P < 0.01, ***P < 0.001. Fig. S2. Hub prognostic ARGs in OC. Uni_cox (A) and Mult_cox (B) for 13 signatures in OC. Overall survival significance confirmed by the nomogram (C) and calibration curve (D). Drug sensitivity analysis for EGF, ANGPTL4, RUNX1, PLG, and NOTCH4 in the CTRP database (E) and GDSC database (F). PCR analysis (G) for the level of ANGPTL4 and ESM1 in SKOV3 treated with different doses of bevacizumab *P < 0.05, **P < 0.01, ***P < 0.001. Fig. S3. The expression of ANGPTL4 in pan-carcinoma based TCGA database and GTEx database. The blue is for normal tissue samples and the red is for cancer samples. Fig. S4. The expression of downstream genes in HeyA8 cells after ANGPTL4 overexpression. A ANGPTL4 expression confirmed in Hey-A8 cells by IF staining. B Volcano plot for DEG expression after ANGPTL4 overexpression by RNA sequencing. C Heatmaps for DEG expression. D GSVA analysis and E KEGG analysis for 14 DEGs. Fig. S5. The level of free ANGPTL4 in CM. Free ANGPTL4 expression confirmed in the CM of SKOV3 and Hey-A8 cells by ELISA. *P < 0.05. Fig. S6. ESM1 was a key factor in the downstream of JAK-STAT pathway. A The expression of ESM1 in SKOV3-DMSO, SKOV3-Colivelin, Hey-A8-DMSO, and Hey-A8-AG490 groups. B The effect of ESM1 on the angiogenesis ability of OC induced by JAK inhibitor/activator. C The effect of ANGPTL4 on ESM1 expression. D Co-IP showed the effects of JAK activator Colivelin on the interaction between ANGPTL4 and ESM1 in SKOV3 cells. Fig. S7. The ANGPTL4/ESM1 axis promotes OC growth and a [file 12967_2023_4819_MOESM1_ESM.zip › Supplementary/Supplementary Fig3.jpg]

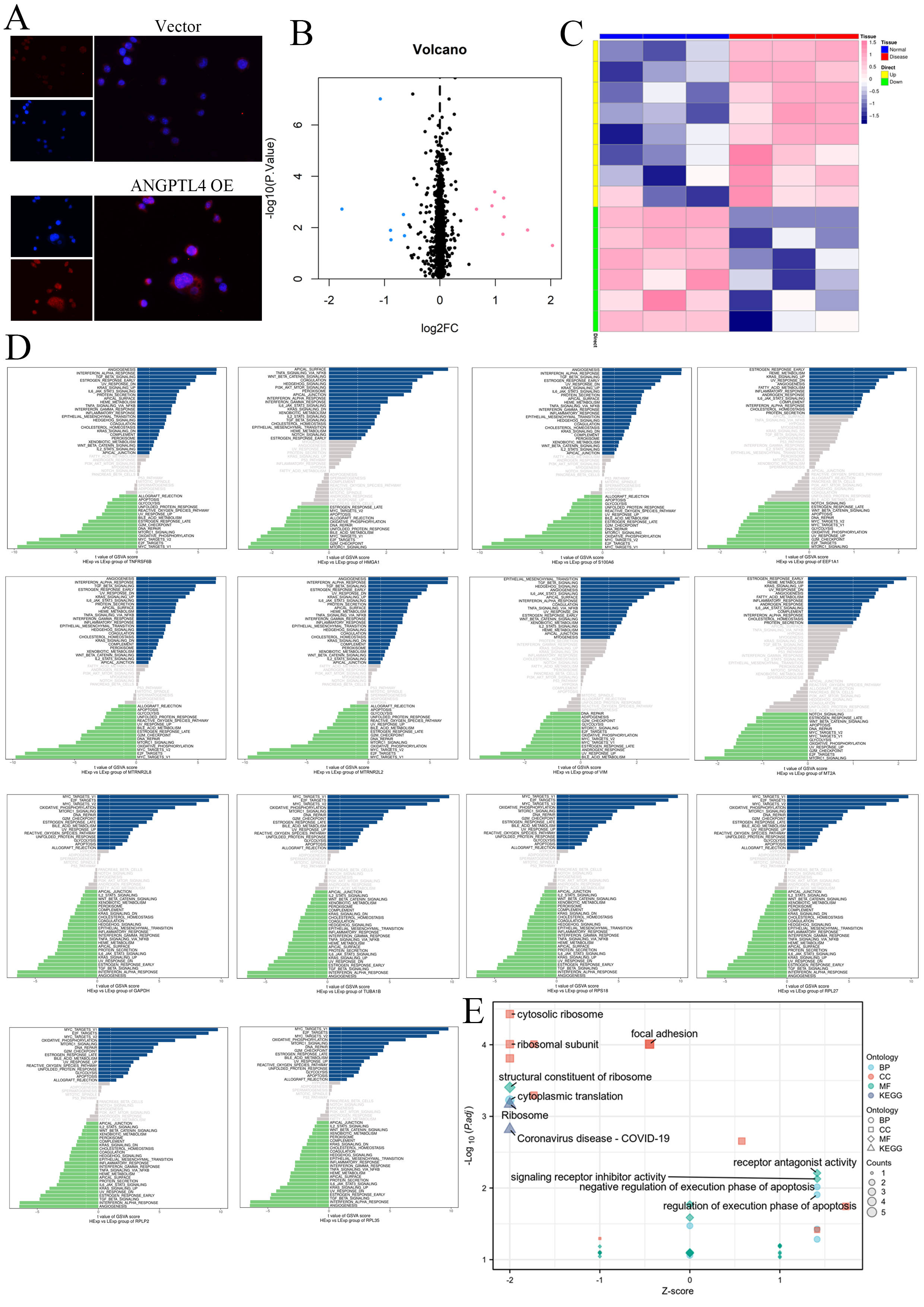

Supplement: Supplementary file 1 — Additional file 1: Fig. S1. Prognostic significance of ARGs in OC patients. A Prognostic models were constructed by LASSO regression based on ARGs. B Lambda on abscissa, coefficients on ordinate. C The survival times, risk score, and signature expression in OC patients. D Overall survival analysis for OC patients with high or low risk. E ROC curves for survival analysis. F Correlation analysis between the risk score and immune cell infiltration. *P < 0.05, **P < 0.01, ***P < 0.001. Fig. S2. Hub prognostic ARGs in OC. Uni_cox (A) and Mult_cox (B) for 13 signatures in OC. Overall survival significance confirmed by the nomogram (C) and calibration curve (D). Drug sensitivity analysis for EGF, ANGPTL4, RUNX1, PLG, and NOTCH4 in the CTRP database (E) and GDSC database (F). PCR analysis (G) for the level of ANGPTL4 and ESM1 in SKOV3 treated with different doses of bevacizumab *P < 0.05, **P < 0.01, ***P < 0.001. Fig. S3. The expression of ANGPTL4 in pan-carcinoma based TCGA database and GTEx database. The blue is for normal tissue samples and the red is for cancer samples. Fig. S4. The expression of downstream genes in HeyA8 cells after ANGPTL4 overexpression. A ANGPTL4 expression confirmed in Hey-A8 cells by IF staining. B Volcano plot for DEG expression after ANGPTL4 overexpression by RNA sequencing. C Heatmaps for DEG expression. D GSVA analysis and E KEGG analysis for 14 DEGs. Fig. S5. The level of free ANGPTL4 in CM. Free ANGPTL4 expression confirmed in the CM of SKOV3 and Hey-A8 cells by ELISA. *P < 0.05. Fig. S6. ESM1 was a key factor in the downstream of JAK-STAT pathway. A The expression of ESM1 in SKOV3-DMSO, SKOV3-Colivelin, Hey-A8-DMSO, and Hey-A8-AG490 groups. B The effect of ESM1 on the angiogenesis ability of OC induced by JAK inhibitor/activator. C The effect of ANGPTL4 on ESM1 expression. D Co-IP showed the effects of JAK activator Colivelin on the interaction between ANGPTL4 and ESM1 in SKOV3 cells. Fig. S7. The ANGPTL4/ESM1 axis promotes OC growth and a [file 12967_2023_4819_MOESM1_ESM.zip › Supplementary/Supplementary Fig4.jpg]

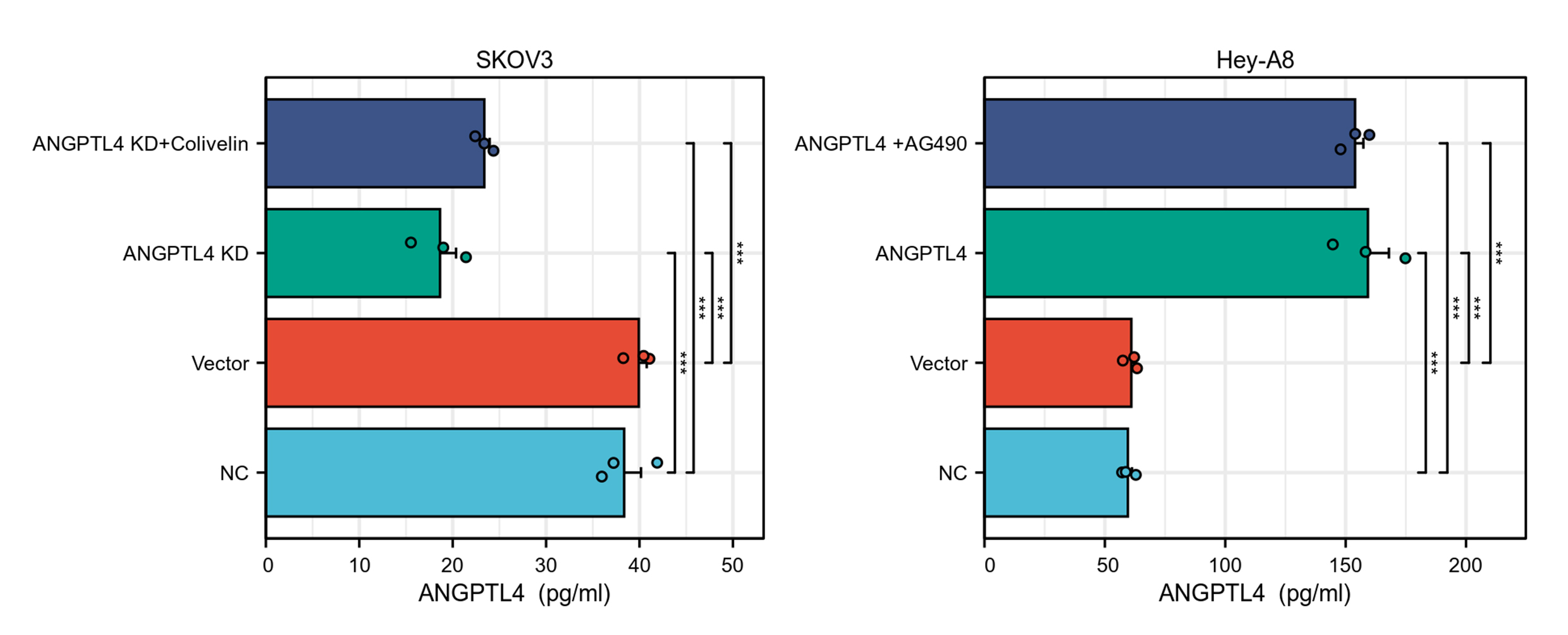

Supplement: Supplementary file 1 — Additional file 1: Fig. S1. Prognostic significance of ARGs in OC patients. A Prognostic models were constructed by LASSO regression based on ARGs. B Lambda on abscissa, coefficients on ordinate. C The survival times, risk score, and signature expression in OC patients. D Overall survival analysis for OC patients with high or low risk. E ROC curves for survival analysis. F Correlation analysis between the risk score and immune cell infiltration. *P < 0.05, **P < 0.01, ***P < 0.001. Fig. S2. Hub prognostic ARGs in OC. Uni_cox (A) and Mult_cox (B) for 13 signatures in OC. Overall survival significance confirmed by the nomogram (C) and calibration curve (D). Drug sensitivity analysis for EGF, ANGPTL4, RUNX1, PLG, and NOTCH4 in the CTRP database (E) and GDSC database (F). PCR analysis (G) for the level of ANGPTL4 and ESM1 in SKOV3 treated with different doses of bevacizumab *P < 0.05, **P < 0.01, ***P < 0.001. Fig. S3. The expression of ANGPTL4 in pan-carcinoma based TCGA database and GTEx database. The blue is for normal tissue samples and the red is for cancer samples. Fig. S4. The expression of downstream genes in HeyA8 cells after ANGPTL4 overexpression. A ANGPTL4 expression confirmed in Hey-A8 cells by IF staining. B Volcano plot for DEG expression after ANGPTL4 overexpression by RNA sequencing. C Heatmaps for DEG expression. D GSVA analysis and E KEGG analysis for 14 DEGs. Fig. S5. The level of free ANGPTL4 in CM. Free ANGPTL4 expression confirmed in the CM of SKOV3 and Hey-A8 cells by ELISA. *P < 0.05. Fig. S6. ESM1 was a key factor in the downstream of JAK-STAT pathway. A The expression of ESM1 in SKOV3-DMSO, SKOV3-Colivelin, Hey-A8-DMSO, and Hey-A8-AG490 groups. B The effect of ESM1 on the angiogenesis ability of OC induced by JAK inhibitor/activator. C The effect of ANGPTL4 on ESM1 expression. D Co-IP showed the effects of JAK activator Colivelin on the interaction between ANGPTL4 and ESM1 in SKOV3 cells. Fig. S7. The ANGPTL4/ESM1 axis promotes OC growth and a [file 12967_2023_4819_MOESM1_ESM.zip › Supplementary/Supplementary Fig5.jpg]

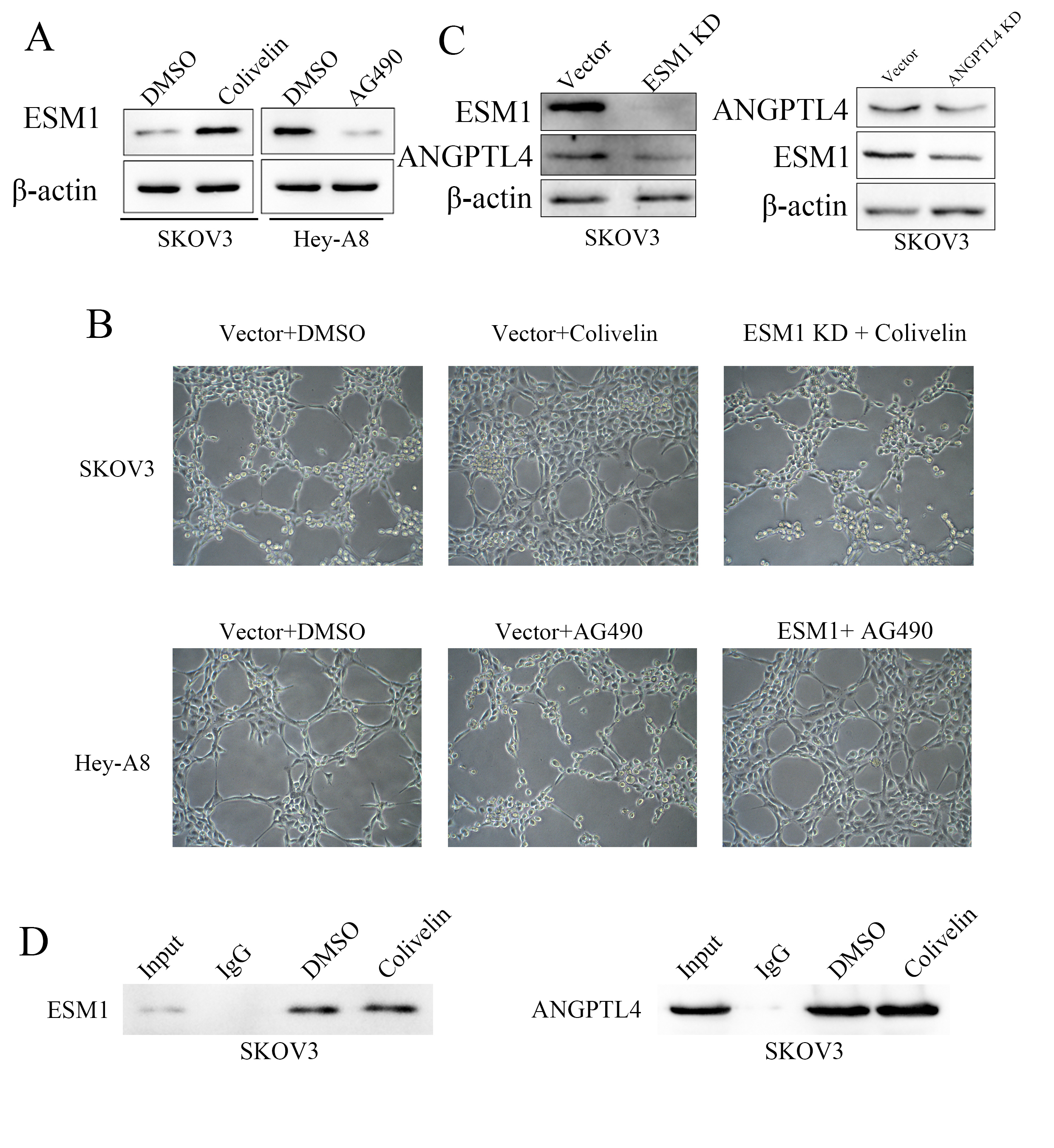

Supplement: Supplementary file 1 — Additional file 1: Fig. S1. Prognostic significance of ARGs in OC patients. A Prognostic models were constructed by LASSO regression based on ARGs. B Lambda on abscissa, coefficients on ordinate. C The survival times, risk score, and signature expression in OC patients. D Overall survival analysis for OC patients with high or low risk. E ROC curves for survival analysis. F Correlation analysis between the risk score and immune cell infiltration. *P < 0.05, **P < 0.01, ***P < 0.001. Fig. S2. Hub prognostic ARGs in OC. Uni_cox (A) and Mult_cox (B) for 13 signatures in OC. Overall survival significance confirmed by the nomogram (C) and calibration curve (D). Drug sensitivity analysis for EGF, ANGPTL4, RUNX1, PLG, and NOTCH4 in the CTRP database (E) and GDSC database (F). PCR analysis (G) for the level of ANGPTL4 and ESM1 in SKOV3 treated with different doses of bevacizumab *P < 0.05, **P < 0.01, ***P < 0.001. Fig. S3. The expression of ANGPTL4 in pan-carcinoma based TCGA database and GTEx database. The blue is for normal tissue samples and the red is for cancer samples. Fig. S4. The expression of downstream genes in HeyA8 cells after ANGPTL4 overexpression. A ANGPTL4 expression confirmed in Hey-A8 cells by IF staining. B Volcano plot for DEG expression after ANGPTL4 overexpression by RNA sequencing. C Heatmaps for DEG expression. D GSVA analysis and E KEGG analysis for 14 DEGs. Fig. S5. The level of free ANGPTL4 in CM. Free ANGPTL4 expression confirmed in the CM of SKOV3 and Hey-A8 cells by ELISA. *P < 0.05. Fig. S6. ESM1 was a key factor in the downstream of JAK-STAT pathway. A The expression of ESM1 in SKOV3-DMSO, SKOV3-Colivelin, Hey-A8-DMSO, and Hey-A8-AG490 groups. B The effect of ESM1 on the angiogenesis ability of OC induced by JAK inhibitor/activator. C The effect of ANGPTL4 on ESM1 expression. D Co-IP showed the effects of JAK activator Colivelin on the interaction between ANGPTL4 and ESM1 in SKOV3 cells. Fig. S7. The ANGPTL4/ESM1 axis promotes OC growth and a [file 12967_2023_4819_MOESM1_ESM.zip › Supplementary/Supplementary Fig6.jpg]

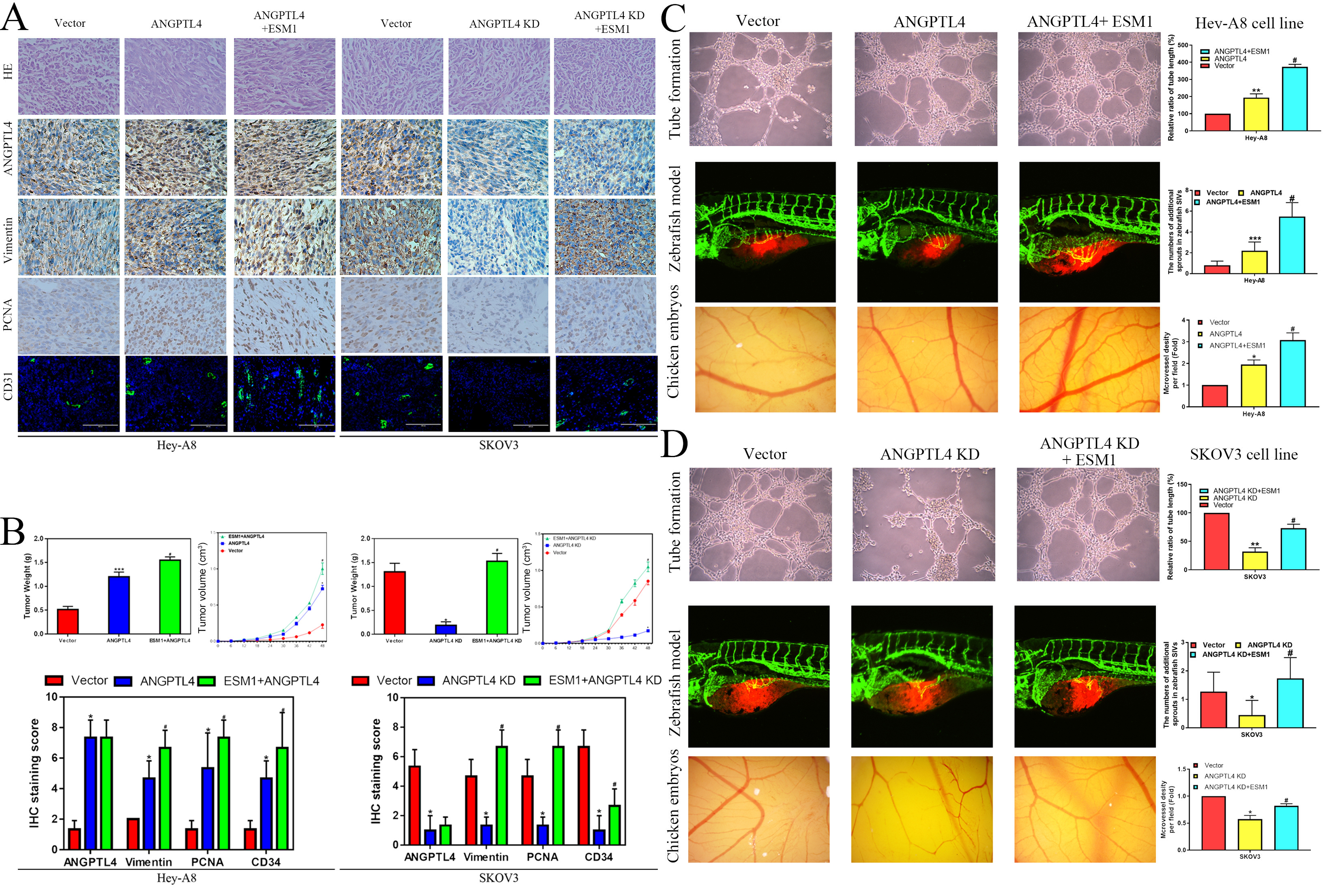

Supplement: Supplementary file 1 — Additional file 1: Fig. S1. Prognostic significance of ARGs in OC patients. A Prognostic models were constructed by LASSO regression based on ARGs. B Lambda on abscissa, coefficients on ordinate. C The survival times, risk score, and signature expression in OC patients. D Overall survival analysis for OC patients with high or low risk. E ROC curves for survival analysis. F Correlation analysis between the risk score and immune cell infiltration. *P < 0.05, **P < 0.01, ***P < 0.001. Fig. S2. Hub prognostic ARGs in OC. Uni_cox (A) and Mult_cox (B) for 13 signatures in OC. Overall survival significance confirmed by the nomogram (C) and calibration curve (D). Drug sensitivity analysis for EGF, ANGPTL4, RUNX1, PLG, and NOTCH4 in the CTRP database (E) and GDSC database (F). PCR analysis (G) for the level of ANGPTL4 and ESM1 in SKOV3 treated with different doses of bevacizumab *P < 0.05, **P < 0.01, ***P < 0.001. Fig. S3. The expression of ANGPTL4 in pan-carcinoma based TCGA database and GTEx database. The blue is for normal tissue samples and the red is for cancer samples. Fig. S4. The expression of downstream genes in HeyA8 cells after ANGPTL4 overexpression. A ANGPTL4 expression confirmed in Hey-A8 cells by IF staining. B Volcano plot for DEG expression after ANGPTL4 overexpression by RNA sequencing. C Heatmaps for DEG expression. D GSVA analysis and E KEGG analysis for 14 DEGs. Fig. S5. The level of free ANGPTL4 in CM. Free ANGPTL4 expression confirmed in the CM of SKOV3 and Hey-A8 cells by ELISA. *P < 0.05. Fig. S6. ESM1 was a key factor in the downstream of JAK-STAT pathway. A The expression of ESM1 in SKOV3-DMSO, SKOV3-Colivelin, Hey-A8-DMSO, and Hey-A8-AG490 groups. B The effect of ESM1 on the angiogenesis ability of OC induced by JAK inhibitor/activator. C The effect of ANGPTL4 on ESM1 expression. D Co-IP showed the effects of JAK activator Colivelin on the interaction between ANGPTL4 and ESM1 in SKOV3 cells. Fig. S7. The ANGPTL4/ESM1 axis promotes OC growth and a [file 12967_2023_4819_MOESM1_ESM.zip › Supplementary/Supplementary Fig7.jpg]
